# Spinal cord injury: global burden from 1990 to 2019 and projections up to 2030 using Bayesian age-period-cohort analysis

**DOI:** 10.3389/fneur.2023.1304153

**Published:** 2023-12-05

**Authors:** Yanbo Liu, Xuesong Yang, Zhigang He, Juan Li, Yijing Li, Yanqiong Wu, Anne Manyande, Maohui Feng, Hongbing Xiang

**Affiliations:** ^1^Department of Anesthesiology and Pain Medicine, Hubei Key Laboratory of Geriatric Anesthesia and Perioperative Brain Health, Wuhan Clinical Research Center for Geriatric Anesthesia, Tongji Hospital, Tongji Medical College, Huazhong University of Science and Technology, Wuhan, China; ^2^School of Human and Social Sciences, University of West London, London, United Kingdom; ^3^Department of Gastrointestinal Surgery, Zhongnan Hospital of Wuhan University, Clinical Medical Research Center of Peritoneal Cancer of Wuhan, Clinical Cancer Study Center of Hubei Provence, Hubei Key Laboratory of Tumor Biological Behaviors, Wuhan, China

**Keywords:** spinal cord injury, risk factor, global incidence, global mortality, global disability, Global Burden of Disease

## Abstract

**Background:**

Spinal cord injuries, often resulting from spine fractures, can lead to severe lifelong symptoms such as paraplegia and even mortality. Over the past few decades, there has been a concerning increase in the annual incidence and mortality rates of spinal cord injuries, which has also placed a growing financial strain on healthcare systems. This review aims to offer a comprehensive overview of spinal cord injuries by estimating their global incidence, prevalence, and the impact in terms of years lived with disability, using data obtained from the 2019 Global Burden of Disease Study.

**Method:**

In this study, we utilized data from the 2019 Global Burden of Disease Study, a widely recognized source for global health data. Our methodology involved estimating the global incidence and prevalence of spinal cord injuries while also assessing the impact on years lived with a disability. We analyzed this data comprehensively to identify patterns and trends and made predictions.

**Finding:**

This research delved into the evolving trends in the global burden of spinal cord injuries, identified key risk factors, and examined variations in incidence and disability across different Socio-demographic Index (SDI) levels and age groups. Briefly, in 2019, the global incidence and burden of YLDs of SCI significantly increased compared to 1990. While males had higher incidence rates compared to females. Falls were identified as the primary cause of SCI. Trend projections up to 2030 revealed a slight decrease in ASIR for males, an upward trend in age-specific incidence rates for both sexes and a similar pattern in age-standardized YLD rates. Additionally, our findings provided crucial groundwork for shaping future policies and healthcare initiatives, with the goal of mitigating the burden of spinal cord injuries, enhancing patient outcomes, and fortifying prevention efforts.

**Interpretation:**

Understanding the global burden of spinal cord injuries is essential for designing effective healthcare policies and prevention strategies. With the alarming increase in prevalence rates and their significant impact on individuals and healthcare systems, this research contributes vital insights to guide future efforts in reducing the incidence of spinal cord injuries, improving the quality of life for affected individuals, and reducing the economic burden on healthcare systems worldwide.

## Introduction

Spinal cord injury is one of the serious complications of spine fracture (1, 2). Acute elevated intraspinal pressure is caused by dislocation or fracture of the vertebral spinal cord or cauda equina and results in various neurological dysfunctions (3–5). The main symptoms of spinal cord injury include spinal cord concussion, incomplete spinal cord injury, complete spinal injury, injury of conus medullaris, and injury of the cauda equina (6, 7). The surgical treatment is based on decompressing the spinal cord and restoring stability (8–10). Nevertheless, non-surgical treatment is equally important. As a result, more research encourages the early application of steroids within 6 h, which can attenuate edema of the spinal cord and prevent injury from worsening (11, 12). Since neuron damage is irreversible, the approach mentioned above can only relieve symptoms and prevent continuous damage.

High-level spinal cord injury refers to damage occurring in the upper region of the spinal cord, typically in the cervical vertebrae area, which can result in various complications such as respiratory dysfunction, bladder control problems, and psychological issues (13–16). Specifically, respiratory dysfunction is a critical and potentially life-threatening condition (17). The phrenic nerve originates from C3-C5, and injuries above such a level can result in diaphragm dysfunction, which directly causes compromised ventilation and respiratory distress. Coughing is crucial for clearing the airways. High cervical spinal cord injuries can weaken the muscles involved in coughing, making it difficult for patients to effectively clear their airways (18). Reduced cough reflex and compromised respiratory function can lead to increased susceptibility to respiratory infections, such as pneumonia (19). Due to restricted sensory and motor functions, patients with spinal cord injuries are also susceptible to pressure ulcers because of prolonged periods lying down. Thus, complications of pressure ulcers can even be life-threatening (20).

As a life-long disease, spinal cord injury brings enormous economic burdens. The estimated annual incidence of spinal injury ranges from 11.5 to 53.4 cases per million inhabitants, while the post-acute spinal cord injury mortality spans from 4.4 to 16.7% (21). According to an investigation from Ontario, Canada, the lifetime cost of spinal cord injury is $336,000 per person and is also higher for patients with high-level spinal cord injury (22). However, previous Global Burden of Disease studies regarding spinal cord injury are too broad and do not provide a precise exposition of spinal injury, which is the most severe type of spinal cord injury.

In order to identify global trends in spinal cord injuries over the past 30 years and provide data to support the formulation of national health policies and resource allocation decisions, we estimated the global incidence, prevalence, and years lived with the disability of spinal cord injury based on the data gained from the 2019 Global Burden of Disease (GBD) Study. We also revealed differences in the incidence of spinal cord injuries between different countries worldwide and developed innovation in public health in this field through a comprehensive analysis of diseases, risk factors, and health trends. Furthermore, we forecasted the disease burden of spinal cord injury up to 2030, which will provide more detailed epidemiological information and enable more rational policies.

## Methods

### Data source

We employed a cross-sectional approach to assess the global burden of spinal cord injury through systematic analysis. We utilized publicly available modeling data and methodologies sourced from the 2019 Global Burden of Diseases (GBD) study, accessible at http://ghdx.healthdata.org/gbd-results-tool. The GBD study, overseen by the World Health Organization (WHO), coordinated by the Institute of Health Metrics and Evaluation, and generously funded by the Bill and Melinda Gates Foundation, provides comprehensive estimates of disease and injury burden. The GBD uses a variety of interrelated metrics to measure the incidence, prevalence, mortality, years of life lost, years lived with a disability and disability-adjusted life years caused by 369 diseases and injuries in 204 countries and regions based on relevant methods introduced elsewhere (23–29). It uses all available disease and injury data from a variety of sources, including civil registration and vital statistics, household surveys and hospital records including published literature, hospital and outpatient records, censuses, household surveys, surveillance data, civil registration and vital statistics, health service utilization, health insurance claims and other sources (26, 30). In the GBD study, there were two types of injuries, cause of injury and nature of injury. Causes of injury are direct physical causes, such as falls and road injuries, while the nature of the injury is the result of the cause, the physical consequences of the cause including the spinal cord injury in our study (31). Therefore, we present incidence, prevalence, and YLD in this study, but not cause-specific mortality or years of life lost. The International Classification of Diseases codes (ICD 9 and ICD 10) were used to define spinal cord injury in the GBD study (32).

### Inclusion and exclusion criteria

The Global Burden of Disease included research from 1990 to 2019 of only spinal cord injury with comprehensive estimates of disease and injury burden, categorized by geographical location, age, sex, and sociodemographic factors. The exclusion criteria stipulated that in order to prevent risk-outcome pairs from entering and exiting the analysis in each GBD cycle, the associated value of p in existing studies should be greater than 0.1. In addition, papers were excluded if they were not relevant to the research question or based on empirical studies. Finally, papers with substantial unexplained heterogeneity between studies were also excluded.

### Statistical analysis

We extracted annual number and rate for spinal cord injury incidence and YLDs from the GBD 2019 by sex, age, region, and country from 1990 to 2019. The specific methodology for estimating spinal cord injury incidence, prevalence and YLDs for GBD 2019 has been described elsewhere (8). Incidence rates were modeled by the Bayesian meta-regression disease model version 2.1 (DisMod-MR 2.1), a meta-regression tool widely used in the GBD. Incidence data throughout the model were for inpatient plus outpatient injuries. Long-term morbidity was defined as a functional status 1 year after the injury that was lower than the functional status at the time of injury, and then long-term morbidity was converted to prevalence using the ordinary differential equation solver used in DisMod. Short-term incidence was converted to prevalence by multiplying the short-term incidence by the duration of the injury and the prevalence estimates were then multiplied by the disability weights for each nature-specific injury to calculate the YLDs disability weights (33). Bayesian meta-regression allows the evaluation of all available morbidity data, disease prevalence, remission and mortality. ASR was calculated by adjusting for population size (per 100,000) and age structure. The Bayesian meta-regression facilitates a comprehensive analysis of all accessible data on disease incidence, encompassing prevalence, rates of remission, and mortality. It computes the Age-Standardized Rate (ASR) by calibrating for the population size on a per 100,000 people and taking into account the age distribution. To derive our estimates, we calculate all estimates and their corresponding 95% uncertainty intervals (UI). For all estimates, a 95% UI (not including 0) indicates statistical significance (34). In this investigation, we extracted age-standardized incidence (ASIR) and years of disability (ASYR) data for spinal cord injuries, stratified by geographical location, age groups, sex, and causative factors directly from the GBD 2019 dataset. To gage temporal trends in spinal cord injury age-standardized rates (ASR) from 1990 to 2019, we computed the estimated annual percentage change. The estimated annual percentage changes (EAPCs) through a regression model that fits the natural logarithm of ASR against calendar years (*y* = α + βx + ε, where *y* = ln(rate), *x* = calendar year, *ε* = error term). The *β* coefficient signifies the ASR trend, with EAPCs and their 95% confidence intervals (CI) derived from the formula 100 × (exp(*β*) − 1) (35).

Furthermore, we conducted Spearman rank-order correlation analysis to elucidate potential associations between the incidence and burden of spinal cord injuries and the Sociodemographic Index (SDI). SDI is a comprehensive measure of social and demographic development, graded from 0 to 1. Based on their SDI values, we categorized 192 countries and regions into low SDI, middle SDI (low-middle, middle, high-middle), or high SDI nations (36, 37).

### Bayesian age-period-cohort analysis

Utilizing the Global Burden of Disease (GBD) data spanning from 1990 to 2019, we embarked on forecasting the disease burden for the period 2020 to 2030. Our methodology involved two key steps: Initially, we gathered data on the incidence and Years Lived with Disability (YLD) for spinal cord injuries across all age brackets (segmented in 5-year intervals) at both global and regional scales for the years 1990 to 2019. Subsequently, applying a specific formula – the ratio of incidence (or YLD) cases to the corresponding rate for all age groups in the same year – we recalculated the corresponding annual total populations (38). Following this, we employed the Bayesian Age-Period-Cohort (BAPC) model to project the disease burden from 2020 to 2030. APC models analyze registry data based on the individual’s age group, the date of the event (period), and the birth cohort of the individual (39). BAPC models operate without relying on parametric assumptions. Bray juxtaposed projections from linear power models with those from both classical and Bayesian APC models, ultimately deduce that the Bayesian APC approach yields more rational forecasts (40). We used the BAPC and INLA packages in the R program for BAPC analysis. All statistical analyses and visualizations were performed using R statistical software (version 4.2.3).

## Results

### Global incidence of spinal cord injury

In 2019, it was estimated that there were 9 million (95% UI 11.1 to 1,810 million) cases of spinal cord injuries worldwide, marking a 52.7% increase compared to estimates in 1990 (Supplementary Table 1). The age-standardized incidence rate in 2019 stood at 11.5 per 100,000. Interestingly, between 1990 and 2019, the age-standardized incidence rate remained relatively stable for both sexes (estimated annual percentage change−0.08 [95% CI−0.23 to 0.08]).

**Figure 1:**
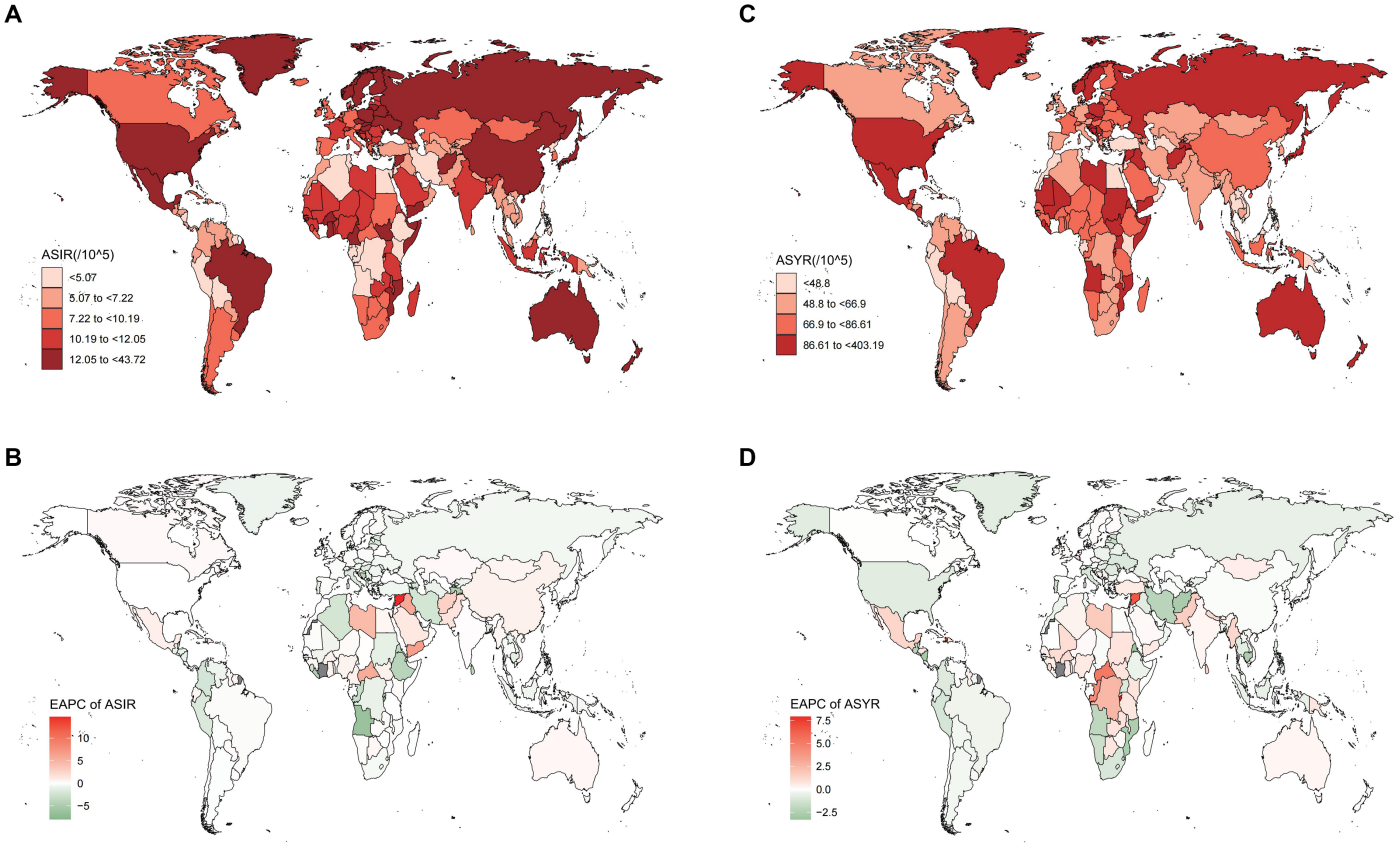
These maps show **(A)** the ASIR and **(C)** the ASYR of spinal cord injury per 100,000 people in 2019 and the EAPC of ASIR **(B)** and ASYR **(D)** from 1990 to 2019 in 204 countries and territories, for both sexes. ASIR, age-standardized incidence rate; ASYR, age-standardized YLD rate; EAPC, estimated annual percentage change.

**Figure 2:**
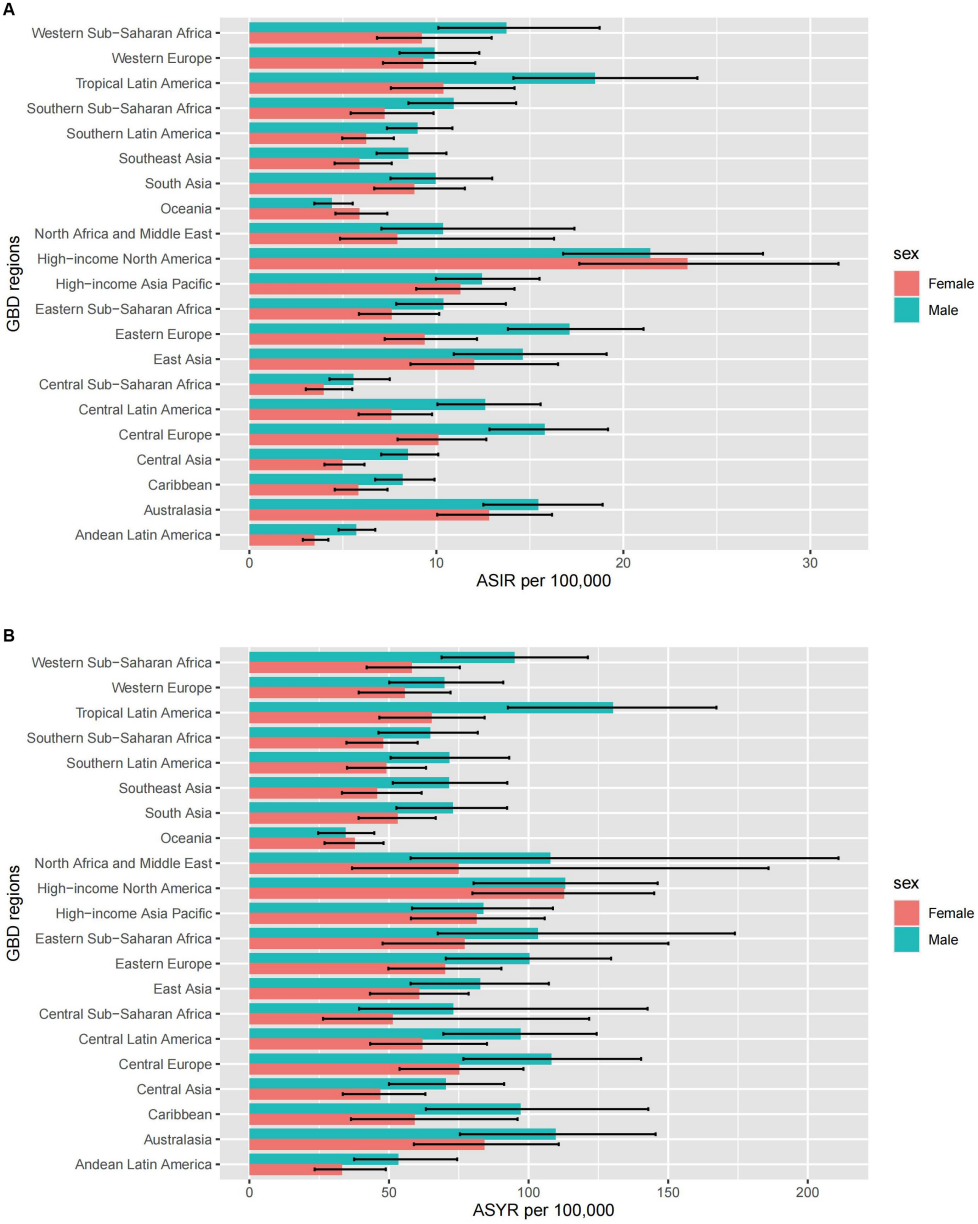
The ASIR **(A)** and ASYR **(B)** due to spinal cord injury by sex, across 21 GBD regions, in 2019. Error bars indicate the 95% uncertainty interval (UI) for the age-standardized rates. ASIR, age-standardized incidence rate; ASYR, age-standardized YLD rate; GBD, Global Burden of Disease.

Afghanistan exhibited the highest age-standardized rate of incidence in 2019 (Figure 1A), while the Syrian Arab Republic showed the most significant increase in age-standardized incidence rates (Figure 1B). On a regional scale, high-income North America reported the highest incidence rate, followed by Tropical Latin America, Australasia, and East Asia (Figure 2A). During the same period, there was a substantial increase in incidence rates in North Africa and the Middle East (estimated annual percentage change 2.2 [95% CI 1.1 to 3.3]), as well as the Caribbean and East Asia (Supplementary Table 1). Conversely, Eastern Sub-Saharan Africa saw a notable decrease (estimated annual percentage change-2.85 [95% CI−4.14 to−1.54]) (Supplementary Table 2).

In terms of standardized incidence rates, the Syrian Arab Republic experienced the highest increase (estimated annual percentage change 14.67 [95% CI 9.88 to 19.68]), while Timor-Leste saw the most substantial decrease (estimated annual percentage change−7.99 [95% CI−10.59 to−5.32]) (Supplementary Table 2). Importantly, some countries with high Sociodemographic Index (SDI), including the Netherlands, Australia, and Canada, reported slight increases in rates (Supplementary Table 2).

### Burden of disease attributable to spinal cord injury

Regionally, mirroring the incidence rate, high-income North America had the highest Years Lived with Disability (YLDs) (Figure 2B). In 2019, YLDs due to spinal cord injury were estimated to be 6.2 million (95% UI 4.5 to 8.2 million) worldwide, marking a 65.4% increase compared to 1990 (Supplementary Table 1). The most significant decrease in YLDs was observed in Andean Latin America (estimated annual percentage change-0.87 [95% CI-0.93 to-0.81]). Despite decreases in most regions, the Caribbean, Central Sub-Saharan Africa, Oceania, and Western Sub-Saharan Africa reported relatively high increased rates (Supplementary Table 2).

Nationally, the Syrian Arab Republic had the highest age-standardized rate of YLDs in 2019, at 403.19 per 100,000 (Supplementary Table 2), followed by Afghanistan and Burundi (Figure 1C). Burundi also showed higher increases in YLD rates than other countries (estimated annual percentage change 7.89 [95% CI 5.07 to 10.79]) (Supplementary Table 2).

### Incidence and disability of spinal cord injury based on SDI, sex, and age

#### Sociodemographic index

**Figure 3:**
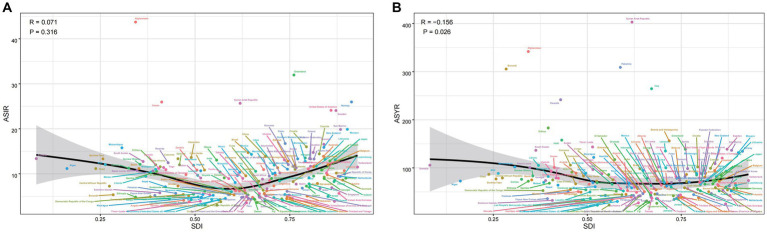
The ASIR **(A)** and ASYR **(B)** of spinal cord injury by 204 countries and territories and sociodemographic index (SDI) in 2019; Expected values are shown as the black line. Each point shows observed ASIR and ASYR for specified country or territory in 2019. ASIR, age-standardized incidence rate; ASYR, age-standardized YLD rate.

**Figure 4:**
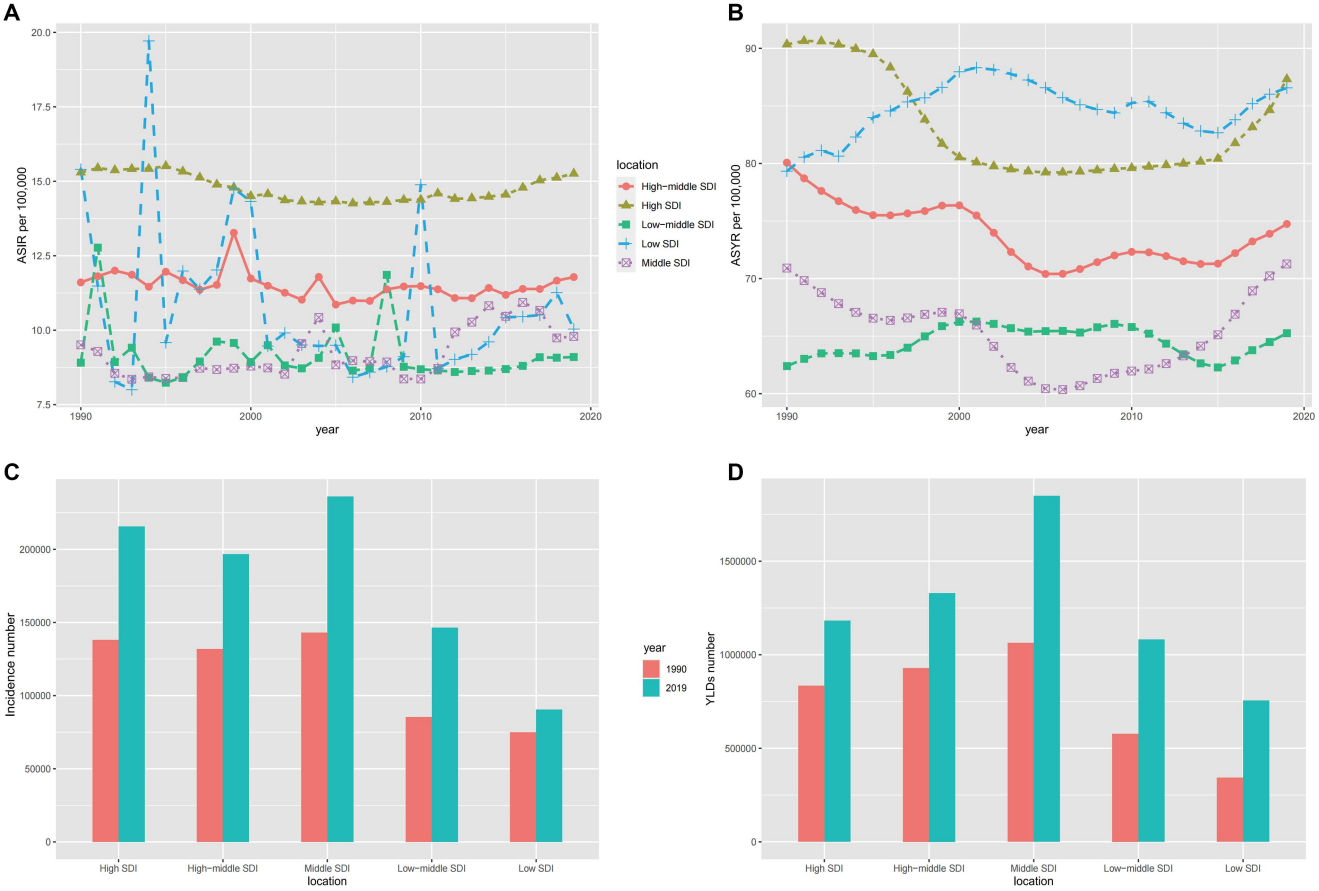
The ASIR **(A)**, ASYR **(B)** and incidence number **(C)**, YLDs number **(D)** for both sexes of spinal cord injury in different SDI-defined regions. SDI: socio-demographic index; ASIR, age-standardized incidence rate; ASYR, age-standardized YLD rate.

In 2019, there was a remarkable trend in the incidence rate with changes in the Sociodemographic Index (SDI) (Figure 3A). However, Spearman rank-order analysis revealed no correlation between age-standardized incidence rates and SDI (rho = 0.071; *p* = 0.316) (Figure 3A). This pattern was also consistent for YLDs (rho = −0.156; *p* < 0.05) (Figure 3B). To gain deeper insights into spinal cord injury epidemiology, we compared the incidence and YLD data across SDI-defined regions. Regions with Middle SDI and Low-Middle SDI had similar Age-Standardized Incidence Rates (ASIR) (Figure 4A). Strikingly, the region with High SDI maintained a consistently high ASIR from 1990 to 2019, while the region with Low SDI saw a shift in ASIR leadership after 1997 (Figure 4B). The region with Middle SDI indicated the highest incidence and YLD numbers in both 1990 and 2019, with substantial increases, while other SDI levels reported lower incidence and moderate increases (Figures 4C,D). In terms of the change in ASYR from 1990 to 2019, high-income North America was even more prominent, with SDI peaking at about 0.78, declining rapidly, and then continuing to rise again (Supplementary Figure 1). In North Africa and the Middle East, ASYR gradually rose with SDI and then rapidly declined. The higher incidence in high SDI regions may be related to a survival bias in these areas, as medical services have enabled the successful resuscitation of injury victims who might have died without treatment, and thus would not have been diagnosed with spinal cord injuries (41).

#### Sex and age

**Figure 5:**
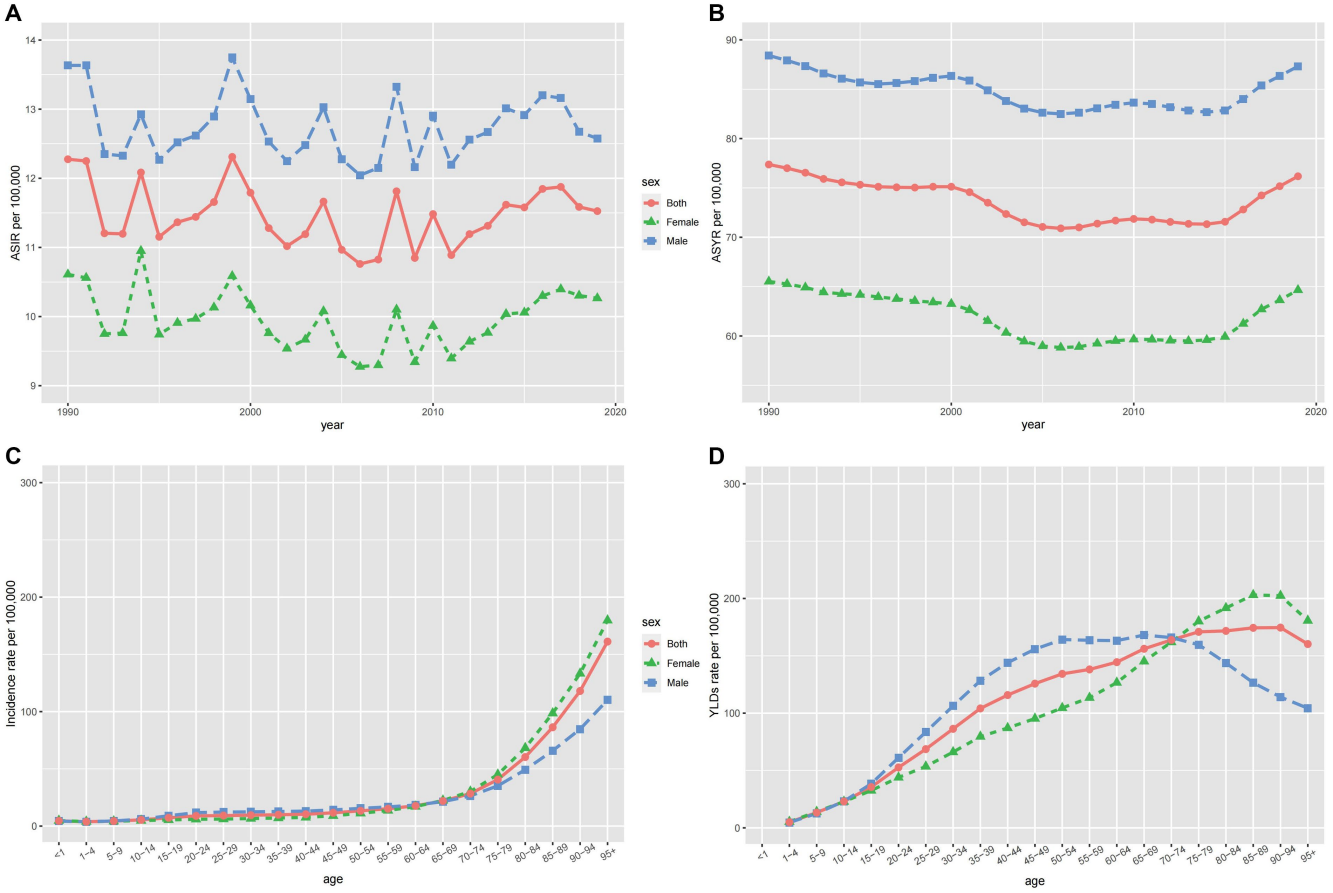
The ASIR **(A)** and ASYR **(B)** of spinal cord injury by sex from 1990 to 2019, and the age-specific numbers of incidence **(C)** and YLDs **(D)** in 2019.

Globally, the age-standardized incidence and YLD rates for women were 10.3 and 64.7 per 100,000, respectively, compared to 12.6 and 87.3 per 100,000 for men in 2019. The gap between men and women persisted from 1990 to 2019 and the burden on men is consistently higher than on women (Figures 5A,B). This may be due to the higher risk of spinal cord injuries in men, as they participate more frequently in social and high-energy sports activities, whereas women are more devoted to household chores and a sedentary lifestyle to protect themselves from harm (42). Among individuals under 70 years of age, incidence rates were similar for both sexes, increasing slightly with age. However, for those older than 70 years, the global incidence rate rose steeply with age (Figure 5C), with women experiencing higher incidence rates. Regarding YLDs, men under 70 years had higher rates than women (Figure 5D), but this pattern reversed in individuals older than 70 years, with women exhibiting higher YLD rates. The reasons might be as follows: On one hand, due to the decline in physical function and health status, older adults people are more prone to falls, which can lead to fractures, spinal cord injuries, or other injuries. On the other hand, the increased prevalence of osteoporosis in postmenopausal women leads to a higher probability of fractures, thereby increasing the incidence of spinal cord injuries.

#### EAPC of spinal cord injury

**Figure 6:**
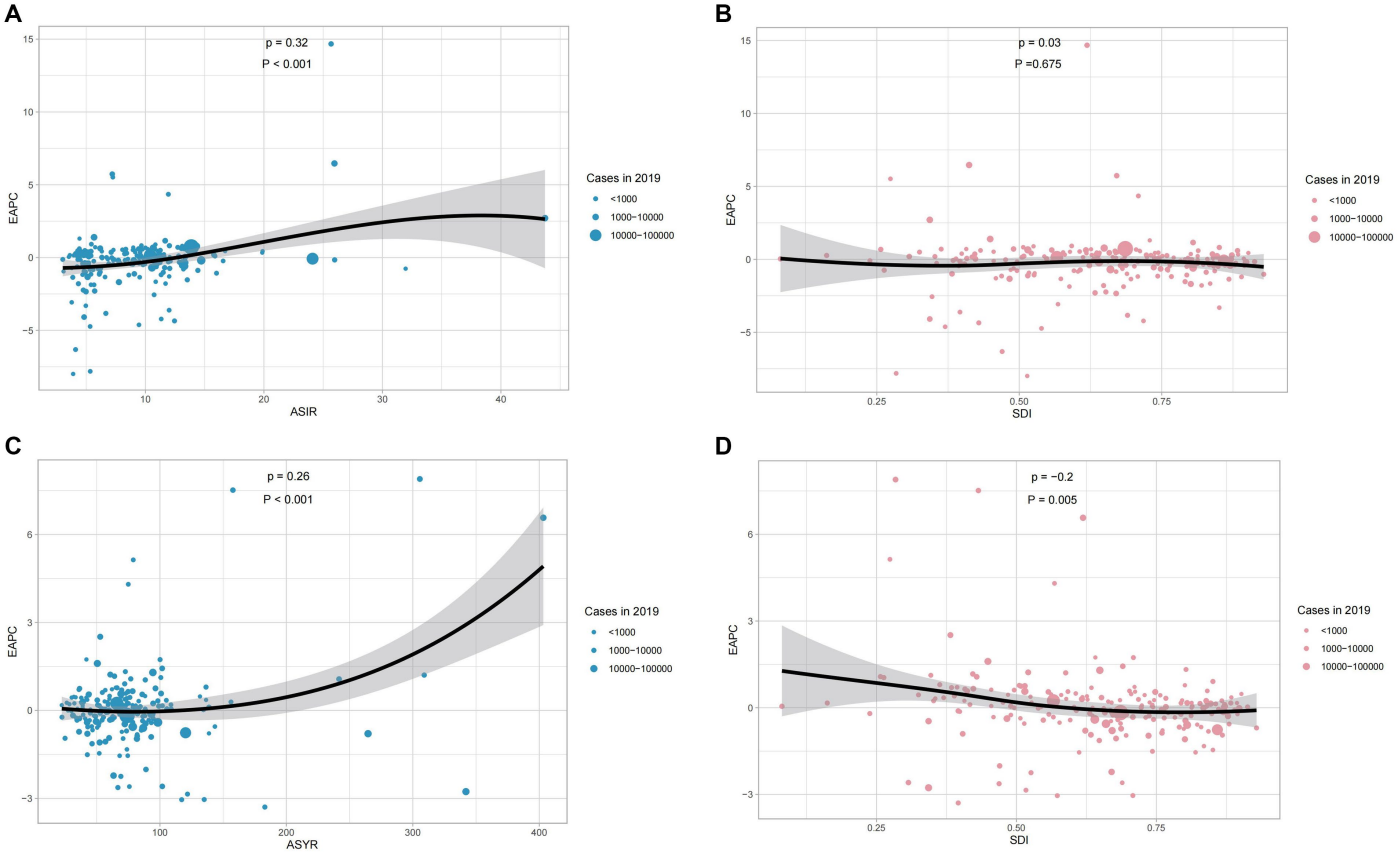
The correlation between EAPC and spinal cord injury ASIR **(A)** and ASYR **(C)** in 2019. The correlation between EAPC of ASIR **(B)** and ASYR **(D)** and SDI in 2019. The circles represent countries that were available on HDI data. The size of circles indicates the number of spinal cord injury patients in 2019. The p stands for Pearson correlation coefficient and the P for *p* values, obtained from Pearson’s correlation analysis. ASIR, age-standardized incidence rate; ASYR, age-standardized YLD rate; EAPC, estimated annual percentage change; SDI, socio-demographic index.

The ASIR increased significantly in North Africa and the Middle East (EAPC = 2.2, 95% CI = 1.1 to 3.3) and decreased remarkably in Eastern Sub − Saharan Africa (EAPC = −2.85, 95% CI = −4.14 to −1.54) (Supplementary Figure 2A; Supplementary Table 1). In the Caribbean, we could see the fastest growth of ASYR which is in contrast to its decline in Andean Latin America (Supplementary Figure 2B). Figure 6 shows the correlation between EAPCs and spinal cord injury ASR [incidence (A), YLDs(C)] in 2019 and HDI [incidence (B), YLDs(D)] in 2019. The circles represent countries that were available on HDI data. The size of circles indicates the number of spinal cord injury patients in 2019. The *p* stands for Pearson correlation coefficient and the P for *p* values, obtained from Pearson’s correlation analysis. Furthermore, EAPC was positively correlated with ASIR (*p* = 0.32, *p* < 0.001), implying that spinal cord injury increased faster in countries with high incidence than in those with low incidence (Figure 6A). However, a correlation between SDI and EAPC was not found (*p* = 0.03, *p* < 0.675) (Figure 6B). Besides, there was a positive correlation between EAPC and ASYR (*p* = 0.26, *p* < 0.001), showing that spinal cord injury increased faster in countries with high Years Lived with a Disability. And there was a weak negative association between EAPC and SDI (Figure 4C, *p* = −0.2, *p* = 0.005) (Figures 6C,D). From 1990 to 2019, the largest ASIR occurred in North Africa and the Middle East. This is because four countries in the region, namely Syria, Yemen, Afghanistan, and Libya, were involved in wars and conflicts during this period. No direct correlation was found between SDI and EAPC suggests that factors other than social development also influence the occurrence of spinal cord injuries. The positive correlation between EAPC and ASYR indicates that in countries with a higher number of disability years, the rate of increase in spinal cord injuries is faster. This highlights the need to pay attention to the long-term impacts of spinal cord injuries and recognize the importance of rehabilitation and supportive treatments.

#### Causes of spinal cord injury

**Figure 7:**
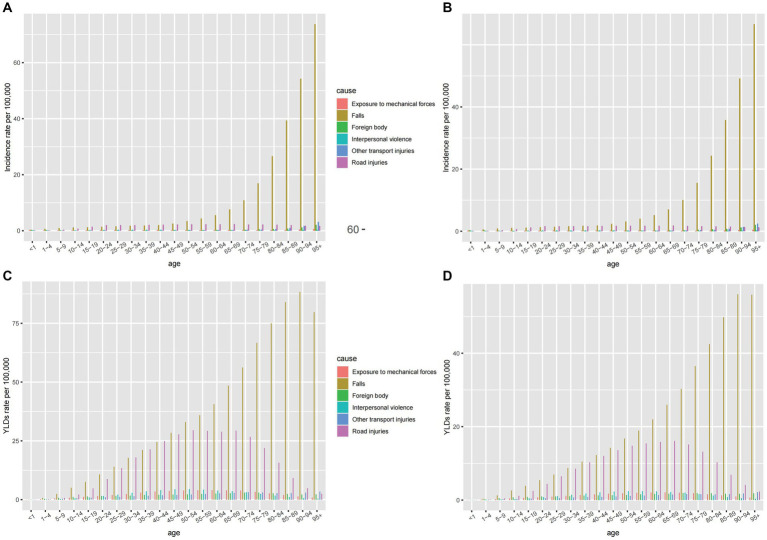
The age-specific rates for incidence of six leading causes of spinal cord injury at neck level **(A)** and below neck level **(B)** in 2019, and the age-specific rates for YLDs of six leading causes of spinal cord injury at neck level **(C)** and below neck level **(D)** in 2019.

**Figure 8:**
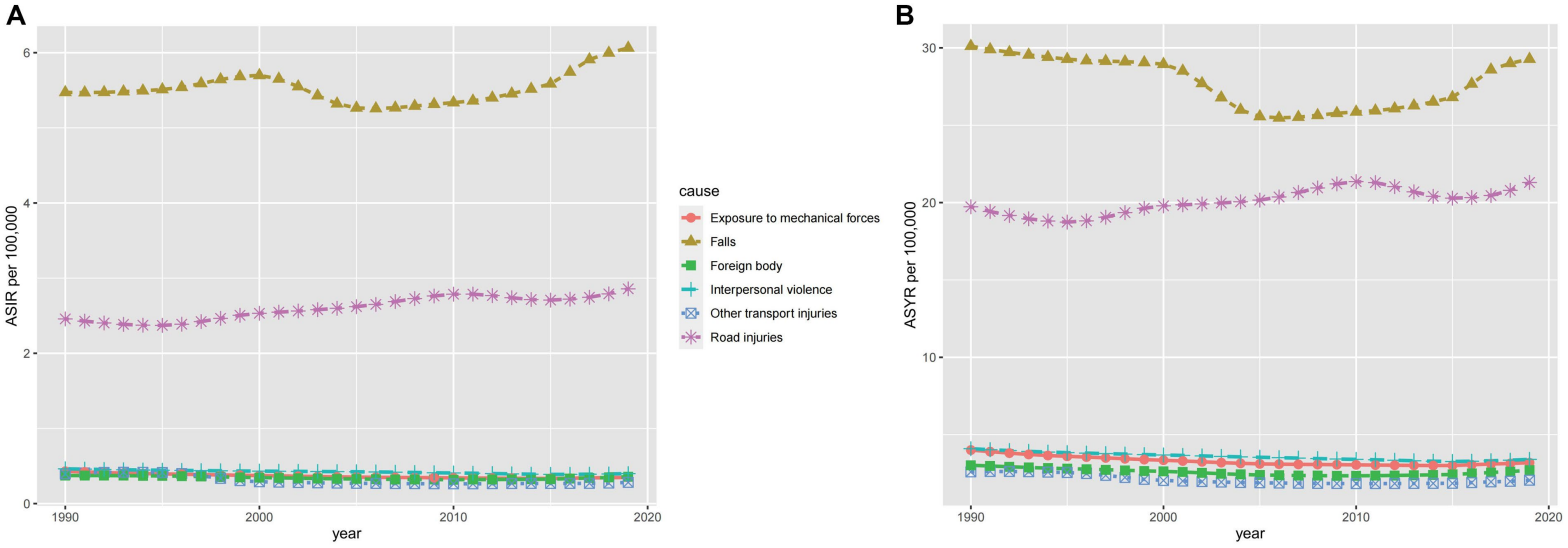
The ASIR **(A)** and ASYR **(B)** of spinal cord injury for the six leading causes from 1990 to 2019. ASIR, age-standardized incidence rate; ASYR, age-standardized YLD rate.

**Figure 9:**
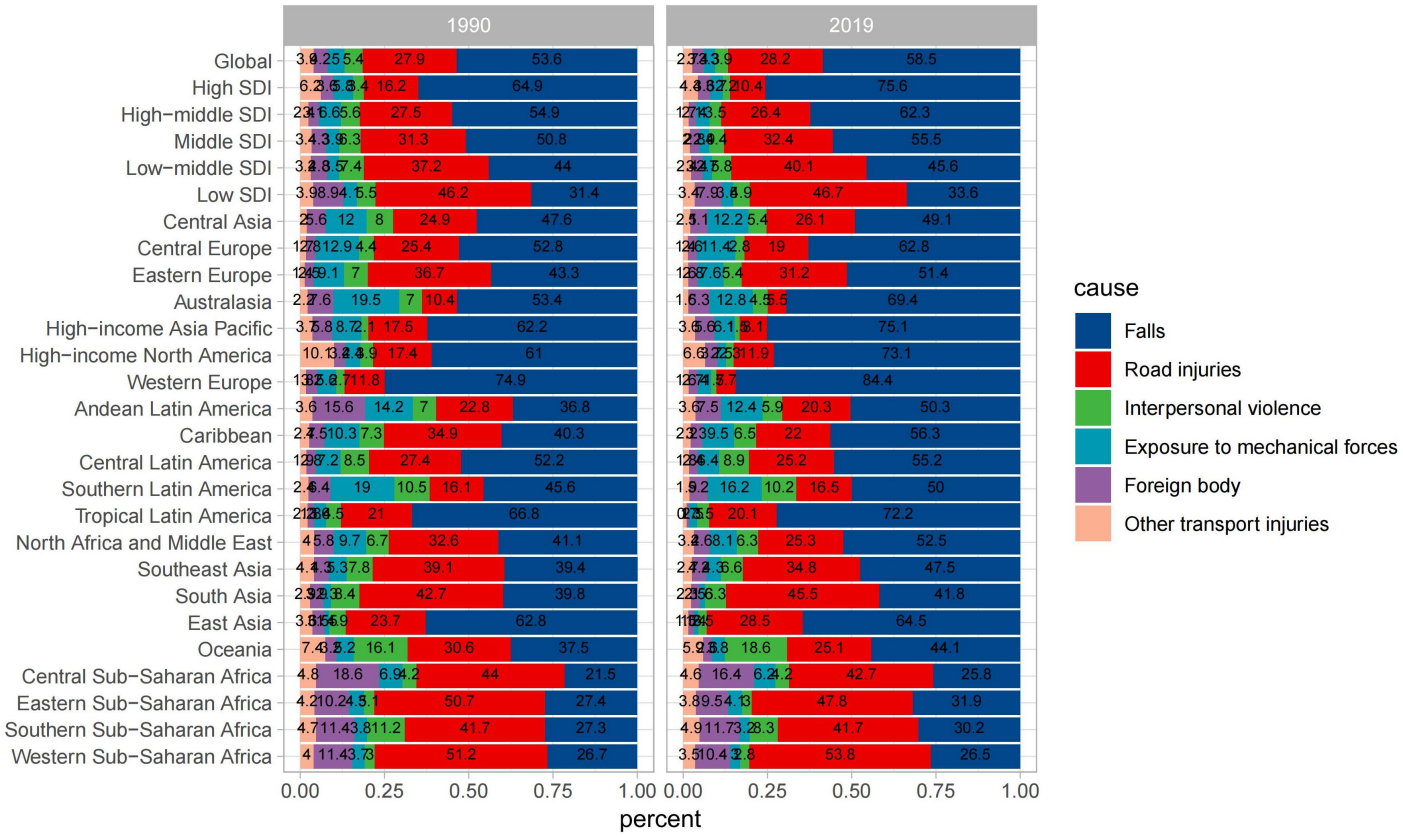
Percentage contributions of major risk factors to ASIR of spinal cord injury, 1990 VS 2019. SDI, Socio-Demographic Index.

The six leading causes of spinal cord injury globally were falls, road injuries, interpersonal violence, exposure to mechanical forces, foreign bodies, and other transport injuries. Figure 7 illustrates these causes, with Figures 7A,C indicating injuries at the neck level and Figures 7B,D representing injuries below the neck. Falls, particularly among the older adults, were the primary cause, followed by road injuries. The incidence due to road injuries increased with age, peaking in the eighties and nineties for both sexes (Figures 7C,D). Figure 8 demonstrates time trends in Age-Standardized Incidence Rates (ASIR) and Age-Standardized Years Lived with Disability Rates (ASYR) due to different risk factors from 1990 to 2019. Falls are consistently ranked as the top risk factor for spinal cord injury, with ASIR and ASYR caused by falls fluctuating over time. ASIR and ASYR from road injuries showed steady increases. Other causes, including exposure to mechanical forces, foreign bodies, interpersonal violence, and other transport injuries, accounted for a smaller fraction, and showed gradual declines. The proportions of spinal cord injury caused by specific causes at the global and regional level in 1990 and 2019 are presented (Figure 9). Globally, more than 50% of spinal cord injuries were caused by falls, followed by road injuries and interpersonal violence. In Western Europe, the proportion of spinal cord injuries caused by falls is even higher than 70%. The proportions remained relatively stable at the global level over time, but in some regions, these significantly changed. For instance, in High-income Asia Pacific, the proportion of falls increased from 62.2% in 1990 to 75.1% in 2016, while that of road injuries decreased from 17.5 to 8.1% during the same period. It is worth mentioning that from 1990 to 2019 the rate of spinal cord injury caused by falls rose in almost all regions except Western Sub-Saharan Africa where it dropped from 26.7 to 26.5%. Falls are the leading cause of spinal cord injuries and are a significant concern as they can be preventable. In younger populations, the risk of falls may be related to environmental factors or other variables and by implementing educational programs and creating safe environmental conditions can help prevent some falls (33). In older adults, the likelihood of falling increases with age and is influenced by various factors. Effective strategies for mitigating falls include screening for conditions such as sarcopenia, vision impairment, and psychiatric disorders, as well as discontinuing psychotropic medications that increase fall risks when appropriate.

#### Spinal cord injuries incidence rate projections up to 2030

**Figure 10:**
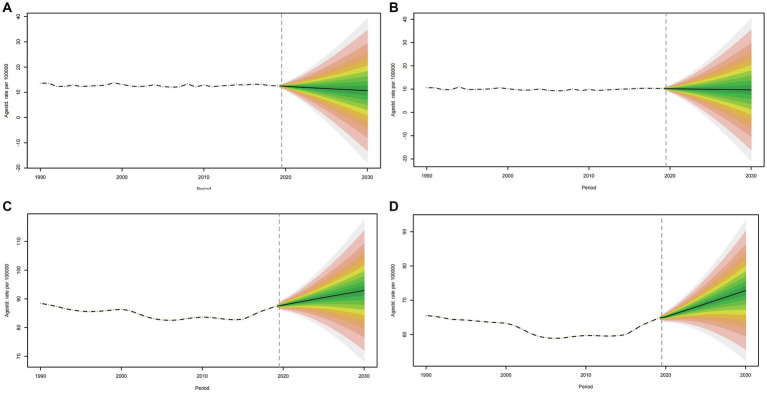
Projections of ASIR **(A, B)** and ASYR **(C, D)** in males and females from 2020 to 2030. The open dot represents the observed value, and the fan shape, the predicted distribution between the 2.5 and 97.5% quantiles. The average forecast is shown as a solid line. The vertical dotted line indicates where the prediction begins. ASIR, age-standardized incidence rate.

The global ASIR for males showed a slight decrease (Figure 10A). For females, the ASIR exhibited a similar pattern to that in males (Figure 10B). However, ASYR, unlike ASIR, revealed an upward trend in both sexes until 2030 (Figures 10C,D). The trend of the age specific incidence rate is the opposite of ASIR for both sexes, and their incidence rate is on the rise in all age groups, peaking in groups aged over 95 years (Supplementary Figures 3, 4). As for the trend of age specific YLDs rate, it has a similar pattern to that of the global ASYR (Supplementary Figures 5, 6). The global ASIR for spinal cord injuries has declined slightly in the future, suggesting that there may be improvements in prevention or changes in exposure to risk factors in the future. The increase in ASYR suggests that the number of years of survival with disability due to spinal cord injury is increasing. This may mean that survival after injury has improved, but that disability will persist.

## Discussion

The systematic analysis presented in this review sheds light on the global incidence, mortality, and disability trends associated with spinal cord injury (SCI) from 1990 to 2019. The research provides insights into the epidemiology of SCI, highlighting both its prevalence and the associated risk factors. Our findings underscore the substantial global burden imposed by SCI, emphasizing its impact on individuals, healthcare systems, and economies.

In 2019, it was estimated that there were 9 million cases of SCI worldwide, representing a 52.7% increase compared to 1990. Furthermore, significant regional disparities in incidence rates were observed, with Afghanistan showing the highest incidence rate, and the Syrian Arab Republic experiencing a notable increase in age-standardized incidence rates. Among high-income regions, North America reported the highest incidence rate. The global burden of Years Lived with Disability (YLDs) due to spinal cord injuries increased by 65.4% from 1990 to 2019. While males had higher incidence rates compared to females, there was a sharp increase in incidence among females aged 70 and above. Falls were identified as the primary cause of SCI. Finally, our review conducted trend projections for the next decade, and revealed a slight decrease in age-standardized incidence rates for males, an upward trend in age-specific incidence rates for both sexes and a similar pattern in age-standardized YLD rates.

The burden of SCI extends beyond its physical manifestations. Patients with SCI are vulnerable to a range of complications that not only affect their quality of life but also pose substantial economic challenges. The study highlights the significant financial burden of SCI, with the lifetime cost estimated at $336,000 per person, a figure that rises for individuals with a high-level of SCI.

Despite the valuable insights we have provided, we do have several limitations. The review relies on data from the Global Burden of Disease Study 2019. While this is a comprehensive source, the accuracy of the data is contingent on the quality of reporting and data collection methods in various regions. Discrepancies in data accuracy and reporting practices between countries may introduce bias. Furthermore, the review identified various causes including falls, road injuries, interpersonal violence, exposure to mechanical forces, foreign bodies, and other transport injuries as leading causes of SCI. While this categorization is informative, it does not delve into the specific circumstances and risk factors associated with each cause. A deeper analysis of the causative factors could inform prevention strategies.

Moreover, the review provides a comprehensive analysis of SCI trends from 1990 to 2019. However, it would be beneficial to explore more recent data to assess whether these trends have continued or evolved since 2019.

## Conclusion

This review offers critical insights into the prevalence, impact, and risk factors associated with SCI. The EAPC of ASYR and ASIR was depicted in this review for the first time, which exhibited the change in age-standard incidence and YLDs. The ASYR and ASIR by sex and SDI in a different GBD region were analyzed. By employing the Bayesian age-period-cohort (BAPC) model, the trends of SCI up to 2030 were projected, and numerous new discoveries were obtained. The results suggest that the global ASIR appears to exhibit a slight decrease in males. In contrast, the ASYR demonstrates a rising trajectory in both sexes until 2030. The trend in age-specific incidence rates opposes that of the ASIR for both males and females, with incidence rates increasing across all age groups, and peaking in the population aged over 95 years. Despite its limitations, this review provides a foundation for further research, policy development, and healthcare initiatives aimed at reducing the burden of SCI, improving patient outcomes, and enhancing prevention efforts. Future studies should strive for more comprehensive and precise data collection and analysis to further advance our understanding of this complex and impactful condition.

## Author contributions

YaL: Formal analysis, Methodology, Software, Visualization, Writing – original draft. XY: Formal analysis, Methodology, Resources, Software, Visualization, Writing – original draft. ZH: Methodology, Writing – original draft. JL: Methodology, Writing – original draft, Writing – review & editing. YiL: Resources, Writing – original draft. YW: Writing – original draft. AM: Writing – review & editing. MF: Funding acquisition, Project administration, Supervision, Validation, Writing – review & editing. HX: Supervision, Validation, Writing – review & editing.
