# Supplementary material for: Spinal cord injury: global burden from 1990 to 2019 and projections up to 2030 using Bayesian age-period-cohort analysis
Source: Front Neurol. 2023 Dec 5;14:1304153. doi: 10.3389/fneur.2023.1304153 (PMC10729761; doi:10.3389/fneur.2023.1304153)
Supplement: Supplementary file 1 [file Table_1.docx]

Table 1. Incidence of Spinal Injuries in 1990 and 2019 for both sexes and all locations, with EAPC from 1990 and 2019.

| location | Num_1990. incidence | Num_2019. incidence | ASR_2019. incidence | EAPC_CI. incidence | Num_1990. YLD | Num_2019. YLD | ASR_2019. YLD | EAPC_CI. YLD |
| --- | --- | --- | --- | --- | --- | --- | --- | --- |
| Global | 595228 (471497 to 766656) | 909001 (706953 to 1156410) | 11.53 (8.97 to 14.67) | -0.08% (-0.23 to 0.08) | 3748672 (2702778 to 4951850) | 6200809 (4465317 to 8156193) | 76.18 (54.77 to 100.37) | -0.19% (-0.29 to -0.09) |
| High SDI | 138117 (109805 to 173946) | 215639 (165052 to 285345) | 15.26 (11.99 to 19.44) | -0.16% (-0.27 to -0.05) | 833957 (593842 to 1070160) | 1182197 (835020 to 1519929) | 87.32 (61.87 to 112.71) | -0.35% (-0.52 to -0.18) |
| High-middle SDI | 131921 (106120 to 164461) | 196775 (150772 to 250908) | 11.78 (9.18 to 14.92) | -0.16% (-0.31 to -0.01) | 929471 (672924 to 1172160) | 1329261 (943002 to 1708999) | 74.74 (53.02 to 96.03) | -0.29% (-0.39 to -0.19) |
| Middle SDI | 143153 (114348 to 179256) | 236077 (182341 to 301998) | 9.79 (7.54 to 12.51) | 0.62% (0.33 to 0.91) | 1063187 (768662 to 1398023) | 1849240 (1325975 to 2468087) | 71.27 (51.21 to 95.66) | -0.14% (-0.35 to 0.06) |
| Low-middle SDI | 85397 (67164 to 110296) | 146465 (115531 to 185659) | 9.1 (7.18 to 11.59) | -0.21% (-0.59 to 0.18) | 577504 (413136 to 796912) | 1081464 (782391 to 1395991) | 65.25 (47.33 to 83.96) | 0.03% (-0.06 to 0.11) |
| Low SDI | 75020 (39852 to 160475) | 90530 (65116 to 134489) | 10.04 (7.37 to 14.46) | -0.71% (-1.56 to 0.16) | 343077 (200435 to 709328) | 755489 (477046 to 1343015) | 86.57 (56.06 to 150.35) | 0.1% (-0.01 to 0.22) |
| Central Sub-Saharan Africa | 3457 (2080 to 6799) | 5318 (3976 to 7773) | 4.76 (3.66 to 6.5) | -2.69% (-4.78 to -0.56) | 23742 (12722 to 50625) | 65208 (33786 to 145484) | 62.11 (33.29 to 134.76) | 0.7% (0.09 to 1.32) |
| East Asia | 104274 (79266 to 134082) | 236241 (173443 to 315144) | 13.49 (9.88 to 18.1) | 0.7% (0.33 to 1.08) | 742342 (531641 to 940603) | 1388823 (979243 to 1793841) | 72.29 (50.95 to 93.5) | -0.17% (-0.58 to 0.23) |
| Eastern Europe | 36775 (29156 to 46344) | 30092 (23743 to 37989) | 13.13 (10.51 to 16.43) | -0.57% (-0.82 to -0.33) | 271646 (192682 to 347314) | 219534 (156030 to 282875) | 84.78 (59.94 to 109.35) | -0.68% (-0.78 to -0.58) |
| Eastern Sub-Saharan Africa | 54804 (22784 to 133741) | 29156 (22453 to 37652) | 8.97 (6.89 to 11.75) | -2.85% (-4.14 to -1.54) | 137377 (79383 to 292137) | 279864 (176063 to 502643) | 90 (57.35 to 160.67) | -0.16% (-0.37 to 0.06) |
| Andean Latin America | 2898 (1837 to 5488) | 2871 (2381 to 3389) | 4.59 (3.83 to 5.43) | -1.05% (-1.55 to -0.56) | 17534 (11289 to 29299) | 26820 (18760 to 38361) | 43.09 (30.14 to 61.58) | -0.87% (-0.93 to -0.81) |
| High-income Asia Pacific | 24139 (19331 to 29846) | 32690 (25123 to 43125) | 11.88 (9.48 to 14.83) | -0.49% (-0.56 to -0.41) | 171547 (120968 to 220202) | 226816 (161070 to 292262) | 82.74 (57.99 to 106.98) | -0.29% (-0.39 to -0.19) |
| High-income North America | 67501 (52610 to 87116) | 114001 (85370 to 155645) | 22.66 (17.44 to 29.89) | -0.08% (-0.27 to 0.1) | 383372 (273104 to 493551) | 543327 (382768 to 696793) | 113.46 (80.1 to 146.02) | -0.73% (-1.08 to -0.38) |
| Caribbean | 2137 (1747 to 2618) | 3459 (2814 to 4227) | 7.06 (5.75 to 8.63) | 0.99% (-1.18 to 3.21) | 14906 (10845 to 18906) | 38262 (24729 to 57334) | 77.87 (49.96 to 117.21) | 2.51% (1.86 to 3.16) |
| Australasia | 2714 (2200 to 3280) | 4996 (3949 to 6243) | 14.2 (11.39 to 17.39) | 0.3% (0.27 to 0.34) | 19732 (13765 to 25937) | 33799 (23765 to 44013) | 96.82 (67.59 to 127.2) | 0.2% (0.16 to 0.24) |
| Central Europe | 19087 (15495 to 23162) | 17227 (13648 to 21504) | 13.04 (10.51 to 15.93) | -1.08% (-1.37 to -0.79) | 134251 (96558 to 170285) | 132314 (94264 to 171655) | 91.84 (65.38 to 119.14) | -0.35% (-0.41 to -0.29) |
| Central Latin America | 19015 (14979 to 24183) | 24749 (19700 to 30982) | 10.03 (7.98 to 12.58) | -0.12% (-0.45 to 0.21) | 152900 (104937 to 229866) | 200134 (142895 to 262800) | 78.68 (56.26 to 103.34) | 0.08% (-0.27 to 0.42) |
| Central Asia | 4887 (4059 to 5797) | 6252 (5146 to 7468) | 6.72 (5.53 to 8.04) | -1.26% (-2.11 to -0.39) | 38774 (28022 to 49649) | 55536 (39430 to 73018) | 58.43 (41.62 to 76.17) | -0.39% (-0.47 to -0.31) |
| North Africa and Middle East | 42700 (28665 to 64843) | 52776 (34780 to 97680) | 9.18 (6 to 17.05) | 2.2% (1.1 to 3.3) | 304143 (139079 to 722558) | 564189 (289631 to 1229092) | 92.17 (47.95 to 198.84) | -0.43% (-0.65 to -0.2) |
| Oceania | 253 (203 to 313) | 569 (466 to 697) | 5.17 (4.21 to 6.3) | -0.37% (-1.55 to 0.82) | 1511 (1114 to 1906) | 4077 (2923 to 5215) | 36.1 (26.06 to 46.09) | 0.55% (0.34 to 0.77) |
| South Asia | 81527 (64240 to 103563) | 156382 (120425 to 201426) | 9.44 (7.29 to 12.09) | 0.05% (-0.28 to 0.38) | 494741 (363959 to 612135) | 1076209 (779951 to 1354932) | 63.17 (46.01 to 79.4) | 0.31% (0.1 to 0.51) |
| Southeast Asia | 36808 (28373 to 48545) | 47473 (37736 to 59770) | 7.27 (5.77 to 9.11) | -0.6% (-1.37 to 0.18) | 258828 (180465 to 372095) | 412470 (297995 to 546543) | 58.58 (42.54 to 77.45) | -0.4% (-0.46 to -0.34) |
| Southern Latin America | 3665 (3016 to 4385) | 5305 (4326 to 6394) | 7.65 (6.26 to 9.23) | -0.02% (-0.07 to 0.03) | 30510 (22019 to 39520) | 43936 (31118 to 56649) | 60.15 (42.45 to 77.74) | -0.25% (-0.3 to -0.19) |
| Southern Sub-Saharan Africa | 4607 (3525 to 6118) | 6590 (5072 to 8666) | 9 (6.96 to 11.84) | -0.27% (-0.45 to -0.09) | 31705 (22957 to 40483) | 41857 (29959 to 52588) | 56.15 (40.2 to 70.59) | -0.84% (-1.18 to -0.5) |
| Tropical Latin America | 21365 (16580 to 27710) | 33477 (25278 to 44097) | 14.44 (10.86 to 19.06) | -0.19% (-0.26 to -0.12) | 146895 (104835 to 186002) | 237248 (169890 to 302343) | 96.84 (69.23 to 123.72) | -0.41% (-0.49 to -0.33) |
| Western Europe | 46448 (36453 to 59098) | 61215 (45912 to 80682) | 9.75 (7.69 to 12.31) | -0.36% (-0.42 to -0.3) | 279889 (197802 to 361524) | 362292 (259168 to 466837) | 62.99 (44.83 to 81.67) | -0.1% (-0.17 to -0.03) |
| Western Sub-Saharan Africa | 16168 (11877 to 22347) | 38162 (28265 to 51944) | 11.37 (8.44 to 15.43) | 0.24% (0.07 to 0.4) | 92325 (67628 to 115005) | 248091 (181695 to 324936) | 75.71 (55 to 97.66) | 0.52% (0.38 to 0.66) |
