# Supplementary material for: Spinal cord injury: global burden from 1990 to 2019 and projections up to 2030 using Bayesian age-period-cohort analysis
Source: Front Neurol. 2023 Dec 5;14:1304153. doi: 10.3389/fneur.2023.1304153 (PMC10729761; doi:10.3389/fneur.2023.1304153)
Supplement: Supplementary file 2 [file Table_2.docx]

Table 2. Incidence of Spinal Injuries in 1990 and 2019 for both sexes in 204 countries, with EAPC from 1990 and 2019.

| location | Num_1990.incidence | Num_2019. incidence | ASR_2019. incidence | EAPC_CI. incidence | Num_1990. YLD | Num_2019. YLD | ASR_2019. YLD | EAPC_CI. YLD |
| --- | --- | --- | --- | --- | --- | --- | --- | --- |
| Afghanistan | 1241 (577 to 2984) | 10452 (3261 to 29635) | 43.72 (11.83 to 131.73) | 2.71% (0.52 to 4.94) | 76762 (9561 to 379) | 98889 (26400 to 287537) | 341.99 (87.3 to 1016.44) | -2.77% (-2.94 to -2.59) |
| Albania | 357 (280 to 449) | 307 (246 to 378) | 10.93 (8.78 to 13.51) | -0.64% (-1.65 to 0.38) | 2910 (2092 to 21) | 3007 (2094 to 3962) | 93.17 (64.84 to 122.9) | -0.42% (-0.59 to -0.25) |
| Algeria | 1191 (957 to 1500) | 1935 (1588 to 2368) | 4.76 (3.89 to 5.84) | -2.24% (-3.29 to -1.17) | 8738 (6214 to 34) | 21542 (14186 to 32871) | 50.46 (33.45 to 75.59) | 0.35% (0.04 to 0.66) |
| American Samoa | 4 (3 to 5) | 5 (4 to 6) | 9.76 (7.54 to 12.6) | 0.56% (-0.64 to 1.77) | 24 (17 to 304) | 37 (27 to 48) | 69.99 (50.29 to 89.75) | 0.56% (0.34 to 0.77) |
| Andorra | 5 (4 to 7) | 12 (9 to 15) | 10.69 (8.35 to 13.58) | 0.05% (0.01 to 0.08) | 38 (27 to 211) | 76 (54 to 99) | 66.89 (47.99 to 87.65) | 0.16% (0.12 to 0.21) |
| Angola | 1919 (702 to 5157) | 971 (759 to 1233) | 4.11 (3.24 to 5.22) | -6.32% (-8.77 to -3.79) | 14892 (5002 to 1014) | 18968 (8592 to 46506) | 88.96 (39.94 to 217.92) | -2.01% (-2.47 to -1.55) |
| Antigua and Barbuda | 3 (2 to 3) | 4 (3 to 5) | 4.89 (3.92 to 5.98) | -0.32% (-1.3 to 0.68) | 21 (15 to 107247) | 39 (28 to 51) | 38.86 (28.03 to 50.11) | 0.1% (-0.06 to 0.26) |
| Argentina | 2425 (2006 to 2895) | 3384 (2755 to 4077) | 7.31 (5.96 to 8.83) | -0.14% (-0.2 to -0.08) | 21112 (15060 to 4529) | 29214 (20449 to 37642) | 60.5 (42.28 to 78.12) | -0.32% (-0.37 to -0.27) |
| Armenia | 294 (243 to 352) | 192 (159 to 231) | 6.21 (5.14 to 7.4) | -0.99% (-1.38 to -0.6) | 5198 (3146 to 3430) | 2673 (1793 to 3841) | 75.85 (51.58 to 108.01) | -2.6% (-2.77 to -2.42) |
| Australia | 2175 (1760 to 2635) | 4146 (3277 to 5191) | 13.89 (11.08 to 17.12) | 0.36% (0.31 to 0.41) | 16039 (11183 to 1005) | 27954 (19462 to 36495) | 94.63 (65.55 to 124.22) | 0.24% (0.19 to 0.29) |
| Austria | 1011 (796 to 1274) | 1209 (922 to 1575) | 9.8 (7.75 to 12.33) | -0.49% (-0.52 to -0.46) | 5901 (4157 to 652) | 7418 (5264 to 9570) | 62.7 (44.15 to 81.43) | -0.21% (-0.25 to -0.16) |
| Azerbaijan | 472 (391 to 570) | 590 (485 to 708) | 5.67 (4.66 to 6.8) | -1.87% (-3.19 to -0.54) | 3604 (2571 to 506) | 6274 (4363 to 8767) | 54.28 (37.87 to 75.18) | -0.33% (-0.5 to -0.15) |
| Bahamas | 11 (9 to 14) | 19 (16 to 23) | 5.05 (4.13 to 6.13) | 0.11% (-0.21 to 0.42) | 84 (60 to 1516) | 158 (113 to 204) | 37.91 (27.22 to 48.88) | 0.25% (0.18 to 0.33) |
| Bahrain | 21 (17 to 27) | 65 (53 to 78) | 4.63 (3.79 to 5.56) | 0.23% (0.05 to 0.4) | 191 (136 to 31842) | 716 (501 to 939) | 39.32 (27.87 to 51.13) | -0.14% (-0.26 to -0.03) |
| Bangladesh | 4734 (3645 to 6205) | 8139 (6241 to 10775) | 5.12 (3.94 to 6.79) | -1.33% (-3.01 to 0.37) | 32876 (23818 to 226) | 77231 (55316 to 100459) | 49.56 (35.51 to 64.32) | -0.38% (-0.69 to -0.06) |
| Barbados | 11 (9 to 13) | 14 (12 to 17) | 4.47 (3.65 to 5.38) | 0.04% (-0.18 to 0.26) | 88 (64 to 510) | 124 (89 to 159) | 34.83 (24.82 to 44.9) | 0.06% (0.01 to 0.11) |
| Belarus | 1441 (1168 to 1785) | 1362 (1099 to 1674) | 12.81 (10.45 to 15.57) | 0.11% (-0.14 to 0.36) | 10900 (7733 to 1121) | 9342 (6561 to 12215) | 78.47 (55.03 to 101.58) | -0.41% (-0.53 to -0.28) |
| Belgium | 1221 (966 to 1531) | 1992 (1500 to 2628) | 12.03 (9.4 to 15.35) | 0.81% (0.52 to 1.1) | 7661 (5439 to 3708) | 10894 (7676 to 14182) | 72.15 (50.85 to 95.41) | 0.91% (0.54 to 1.27) |
| Belize | 11 (8 to 19) | 21 (17 to 26) | 5.32 (4.33 to 6.52) | -0.84% (-1.43 to -0.24) | 59 (42 to 142) | 173 (126 to 226) | 45.01 (32.81 to 58.19) | 0.03% (-0.11 to 0.17) |
| Benin | 367 (272 to 500) | 1004 (729 to 1385) | 11.16 (8.17 to 15.37) | 0.19% (0.16 to 0.23) | 2223 (1613 to 2836) | 6408 (4564 to 8217) | 75.02 (54.02 to 95.02) | 0.24% (0.13 to 0.35) |
| Bermuda | 6 (5 to 8) | 9 (7 to 12) | 11.57 (8.95 to 14.75) | 0.23% (0.12 to 0.33) | 50 (36 to 6) | 69 (49 to 89) | 80.89 (57.53 to 104.76) | 0.17% (0.05 to 0.29) |
| Bhutan | 29 (22 to 39) | 52 (41 to 68) | 7.3 (5.7 to 9.3) | 0.52% (-0.24 to 1.29) | 201 (144 to 4832) | 399 (292 to 506) | 54.72 (40.11 to 69.43) | 0.62% (0.51 to 0.73) |
| Bolivia (Plurinational State of) | 249 (208 to 292) | 487 (405 to 572) | 4.3 (3.57 to 5.06) | -0.15% (-0.29 to -0.01) | 1861 (1329 to 1257) | 3779 (2690 to 4811) | 34.28 (24.44 to 43.53) | -0.32% (-0.38 to -0.27) |
| Bosnia and Herzegovina | 685 (488 to 1106) | 395 (315 to 483) | 11.34 (9.17 to 13.82) | -4.22% (-6.73 to -1.64) | 4834 (3424 to 955) | 6377 (3687 to 12037) | 156.2 (89.93 to 302.34) | 0.29% (-0.54 to 1.12) |
| Botswana | 93 (73 to 120) | 201 (155 to 267) | 9.56 (7.34 to 12.66) | 0.35% (0.28 to 0.41) | 561 (412 to 2895) | 1274 (907 to 1617) | 56.79 (40.57 to 71.74) | 0.69% (0.19 to 1.2) |
| Brazil | 21177 (16415 to 27463) | 33118 (24962 to 43693) | 14.71 (11.05 to 19.43) | -0.19% (-0.26 to -0.12) | 145402 (103654 to 1054) | 234431 (167899 to 298702) | 98.37 (70.3 to 125.71) | -0.4% (-0.48 to -0.33) |
| Brunei Darussalam | 19 (16 to 23) | 32 (26 to 38) | 7.86 (6.43 to 9.45) | -0.37% (-0.45 to -0.29) | 127 (90 to 6) | 234 (164 to 302) | 50.39 (35.42 to 64.88) | -0.58% (-0.67 to -0.5) |
| Bulgaria | 1161 (950 to 1404) | 858 (692 to 1043) | 11.94 (9.69 to 14.47) | -0.3% (-0.37 to -0.23) | 9803 (6941 to 249) | 7357 (5223 to 9532) | 86.29 (61.09 to 111.69) | -0.36% (-0.44 to -0.27) |
| Burkina Faso | 704 (520 to 961) | 2207 (1612 to 3054) | 13.31 (9.75 to 18.34) | 0.68% (0.49 to 0.87) | 3910 (2830 to 120) | 11871 (8646 to 14957) | 76.94 (55.78 to 96.42) | 1.08% (0.82 to 1.34) |
| Burundi | 237 (182 to 307) | 521 (402 to 674) | 5.35 (4.18 to 6.94) | -7.81% (-12.68 to -2.68) | 1295 (929 to 11) | 29623 (9258 to 84723) | 305.56 (98.22 to 849.1) | 7.89% (5.07 to 10.79) |
| Cabo Verde | 26 (20 to 34) | 57 (43 to 76) | 10.84 (8.29 to 14.38) | 0.89% (0.83 to 0.96) | 172 (123 to 15778) | 381 (274 to 483) | 72.2 (51.62 to 91.88) | 0.38% (0.31 to 0.45) |
| Cambodia | 574 (386 to 1005) | 797 (647 to 973) | 5.41 (4.34 to 6.7) | -1.14% (-1.86 to -0.42) | 13370 (3463 to 786) | 10238 (5454 to 22190) | 66.77 (35.46 to 145.55) | -2.63% (-2.7 to -2.56) |
| Cameroon | 879 (632 to 1227) | 2960 (2102 to 4128) | 13.64 (9.78 to 18.86) | 0.71% (0.57 to 0.85) | 5866 (4309 to 1281) | 17898 (12839 to 23285) | 83.11 (60.16 to 105.99) | 0.05% (-0.18 to 0.29) |
| Canada | 2827 (2314 to 3453) | 5431 (4270 to 6912) | 10.16 (8.22 to 12.51) | 0.28% (0.25 to 0.31) | 16435 (11771 to 570) | 27394 (19406 to 35536) | 54.16 (38.05 to 70.21) | 0.05% (0.02 to 0.07) |
| Central African Republic | 83 (67 to 104) | 365 (208 to 741) | 7.25 (4.47 to 13.69) | 5.52% (3.28 to 7.8) | 469 (339 to 702) | 4095 (1737 to 10241) | 78.93 (36.25 to 189.19) | 5.13% (4.04 to 6.24) |
| Chad | 620 (398 to 1058) | 1275 (939 to 1744) | 11.16 (8.21 to 15.24) | -0.1% (-0.65 to 0.47) | 4323 (2513 to 101) | 8833 (6084 to 13034) | 86.55 (60.96 to 126.69) | -0.2% (-0.31 to -0.09) |
| Chile | 965 (798 to 1153) | 1599 (1294 to 1955) | 8.25 (6.73 to 10.04) | 0.26% (0.11 to 0.41) | 7238 (5210 to 1066) | 12222 (8740 to 15961) | 58.6 (42.13 to 76.4) | -0.06% (-0.25 to 0.13) |
| China | 102622 (77801 to 132131) | 234191 (171837 to 312871) | 13.87 (10.15 to 18.66) | 0.73% (0.35 to 1.1) | 731470 (523591 to 1713) | 1373726 (968118 to 1774815) | 74.01 (52.16 to 95.72) | -0.17% (-0.58 to 0.24) |
| Colombia | 3049 (2237 to 4529) | 2768 (2213 to 3431) | 5.72 (4.58 to 7.05) | -2.3% (-2.74 to -1.85) | 21868 (15273 to 23) | 30489 (20572 to 44447) | 59.85 (40.39 to 87.39) | -0.94% (-1.24 to -0.63) |
| Comoros | 49 (35 to 68) | 86 (62 to 122) | 13.35 (9.62 to 18.68) | -0.11% (-0.3 to 0.08) | 334 (246 to 135) | 678 (490 to 855) | 105.73 (76.56 to 132.83) | 0.06% (-0.08 to 0.2) |
| Congo | 79 (62 to 101) | 167 (131 to 213) | 3.78 (2.99 to 4.81) | -3.07% (-6.51 to 0.49) | 460 (331 to 8) | 3607 (1642 to 8799) | 74.98 (35.46 to 177.4) | 4.3% (2.1 to 6.56) |
| Cook Islands | 2 (2 to 3) | 2 (2 to 3) | 11.02 (8.29 to 14.42) | -0.47% (-1.73 to 0.8) | 11 (8 to 1) | 15 (10 to 19) | 71.55 (50.34 to 93.22) | -0.11% (-0.26 to 0.04) |
| Costa Rica | 172 (140 to 211) | 303 (246 to 371) | 6.35 (5.15 to 7.77) | -0.04% (-0.14 to 0.07) | 1189 (848 to 6) | 2299 (1621 to 3010) | 45.36 (31.87 to 59.38) | -0.03% (-0.12 to 0.05) |
| Côte d'Ivoire | 934 (689 to 1281) | 2179 (1598 to 2998) | 11.56 (8.5 to 15.83) | 0.06% (-0.22 to 0.35) | 5561 (3972 to 815) | 14737 (10567 to 18642) | 74.84 (54.01 to 93.77) | 0.64% (0.46 to 0.82) |
| Croatia | 832 (670 to 1027) | 952 (726 to 1242) | 16 (12.63 to 20.31) | -1.07% (-1.77 to -0.38) | 5550 (3949 to 1527) | 5690 (4019 to 7402) | 101.96 (72.63 to 133.65) | 0.16% (0.06 to 0.26) |
| Cuba | 773 (629 to 944) | 1263 (985 to 1628) | 8.34 (6.7 to 10.35) | 0.57% (0.51 to 0.64) | 5056 (3569 to 944) | 6504 (4662 to 8469) | 44.95 (32.13 to 58.67) | -0.09% (-0.17 to -0.01) |
| Cyprus | 67 (54 to 81) | 135 (105 to 173) | 9.28 (7.32 to 11.7) | 0.13% (0.05 to 0.22) | 461 (331 to 3953) | 943 (661 to 1242) | 58.98 (41.29 to 78.06) | 0.19% (0.13 to 0.24) |
| Czechia | 2133 (1690 to 2694) | 1845 (1461 to 2299) | 14.43 (11.66 to 17.75) | -0.88% (-0.96 to -0.8) | 12202 (8618 to 58) | 13522 (9535 to 17554) | 99.1 (69.97 to 129.62) | 0.15% (0.04 to 0.25) |
| Democratic People's Republic of Korea | 662 (539 to 821) | 873 (686 to 1129) | 3.03 (2.41 to 3.88) | -0.13% (-0.4 to 0.15) | 4753 (3395 to 40) | 7008 (5007 to 9073) | 22.56 (16.11 to 29.02) | -0.23% (-0.38 to -0.08) |
| Democratic Republic of the Congo | 1324 (1018 to 1742) | 3704 (2691 to 5623) | 4.91 (3.73 to 7.02) | -0.99% (-3.29 to 1.36) | 7603 (5389 to 3196) | 37780 (20750 to 78035) | 52.78 (30.22 to 104.11) | 2.51% (1.63 to 3.41) |
| Denmark | 752 (577 to 978) | 657 (505 to 851) | 8.6 (6.78 to 10.81) | -1.2% (-1.31 to -1.09) | 3584 (2531 to 175) | 4168 (2926 to 5448) | 57.61 (39.99 to 75.47) | -0.07% (-0.16 to 0.02) |
| Djibouti | 61 (40 to 98) | 119 (88 to 161) | 12.01 (8.97 to 16.29) | -1.3% (-2.12 to -0.46) | 297 (215 to 98) | 952 (678 to 1230) | 91.17 (65.02 to 117.35) | -0.32% (-0.46 to -0.19) |
| Dominica | 3 (2 to 4) | 3 (3 to 4) | 4.44 (3.59 to 5.43) | 1.3% (0.19 to 2.42) | 24 (17 to 173) | 32 (23 to 42) | 41.83 (30 to 54.38) | 0.44% (0.35 to 0.53) |
| Dominican Republic | 282 (222 to 358) | 587 (460 to 736) | 5.44 (4.29 to 6.83) | 0.92% (0.6 to 1.24) | 2235 (1614 to 8043) | 4755 (3410 to 6125) | 44.27 (31.83 to 56.93) | 0.56% (0.43 to 0.69) |
| Ecuador | 429 (362 to 506) | 912 (763 to 1078) | 5.32 (4.45 to 6.31) | 0.38% (0.13 to 0.63) | 3868 (2730 to 276) | 7139 (5143 to 9248) | 41.84 (30.11 to 54.17) | -0.41% (-0.48 to -0.35) |
| Egypt | 1923 (1536 to 2429) | 4060 (3281 to 5084) | 4.31 (3.49 to 5.41) | 0.45% (0.1 to 0.8) | 14451 (10269 to 11) | 28971 (20531 to 37521) | 31.24 (22.25 to 40.28) | -0.17% (-0.3 to -0.04) |
| El Salvador | 943 (445 to 2252) | 413 (332 to 515) | 6.6 (5.3 to 8.17) | -1.34% (-2.35 to -0.32) | 14593 (4794 to 13) | 7025 (3524 to 16079) | 117.22 (57.73 to 271.86) | -3.04% (-3.08 to -3) |
| Equatorial Guinea | 14 (11 to 17) | 45 (35 to 57) | 3.96 (3.11 to 5.06) | 0.15% (-0.03 to 0.34) | 80 (57 to 27463) | 293 (209 to 379) | 26.36 (19.04 to 33.58) | 0.26% (0.18 to 0.34) |
| Eritrea | 3433 (819 to 10266) | 635 (467 to 861) | 12 (8.95 to 16.28) | -3.61% (-6.28 to -0.87) | 12654 (3458 to 545) | 9577 (4639 to 23553) | 182.99 (89.92 to 457.91) | -3.29% (-3.45 to -3.13) |
| Estonia | 274 (223 to 339) | 160 (128 to 195) | 10.87 (8.86 to 13.19) | -1.66% (-1.78 to -1.54) | 1886 (1327 to 380) | 1225 (867 to 1610) | 73.92 (51.74 to 97.1) | -1.33% (-1.45 to -1.2) |
| Eswatini | 50 (40 to 65) | 90 (69 to 119) | 9.1 (6.99 to 12.02) | 0.48% (0.35 to 0.6) | 321 (232 to 1742) | 514 (364 to 646) | 51.55 (36.52 to 64.56) | -0.52% (-1.06 to 0.03) |
| Ethiopia | 38357 (10931 to 107247) | 4139 (3323 to 5096) | 4.83 (3.84 to 6) | -4.09% (-6.7 to -1.4) | 34524 (16633 to 1879) | 56868 (30373 to 121962) | 72.21 (38.93 to 155.39) | -0.46% (-0.86 to -0.06) |
| Fiji | 23 (19 to 29) | 31 (26 to 38) | 3.62 (2.98 to 4.4) | 0.16% (-0.3 to 0.63) | 172 (123 to 821) | 261 (188 to 336) | 28.43 (20.53 to 36.65) | 0.21% (0.11 to 0.31) |
| Finland | 800 (635 to 1014) | 995 (754 to 1300) | 12.86 (10.07 to 16.37) | -0.32% (-0.65 to 0.01) | 4795 (3379 to 286) | 6140 (4337 to 8023) | 84.54 (59.05 to 111.69) | 0.13% (-0.19 to 0.46) |
| France | 7961 (6227 to 10235) | 10840 (8157 to 14346) | 10.84 (8.54 to 13.75) | -0.31% (-0.35 to -0.27) | 44198 (31606 to 315) | 58574 (41243 to 76394) | 66.91 (46.99 to 88.12) | -0.01% (-0.05 to 0.03) |
| Gabon | 37 (29 to 48) | 67 (53 to 86) | 4.42 (3.47 to 5.63) | -0.15% (-0.23 to -0.06) | 237 (173 to 2694) | 465 (335 to 599) | 29.87 (21.82 to 38.2) | -0.01% (-0.14 to 0.11) |
| Gambia | 65 (49 to 87) | 170 (127 to 229) | 10.5 (7.79 to 14.24) | 0.39% (0.29 to 0.49) | 487 (342 to 2429) | 1107 (798 to 1405) | 68.12 (49.67 to 86.18) | -0.14% (-0.24 to -0.03) |
| Georgia | 455 (375 to 545) | 343 (280 to 418) | 8.95 (7.32 to 10.78) | -0.47% (-1.6 to 0.66) | 3591 (2566 to 29) | 3053 (2133 to 4063) | 73.07 (50.45 to 97.59) | 0.58% (0.42 to 0.74) |
| Germany | 8658 (6811 to 10987) | 11418 (8599 to 15121) | 9.03 (7.09 to 11.34) | -0.2% (-0.33 to -0.07) | 53226 (38070 to 2) | 65939 (46825 to 86203) | 57.56 (40.34 to 76.25) | -0.02% (-0.11 to 0.08) |
| Ghana | 1226 (880 to 1713) | 3340 (2335 to 4766) | 13.04 (9.33 to 18.38) | 0.64% (0.56 to 0.72) | 7883 (5582 to 10266) | 22174 (15891 to 28290) | 85.99 (61.71 to 108.78) | 0.55% (0.43 to 0.67) |
| Greece | 912 (738 to 1121) | 897 (711 to 1125) | 7.27 (5.85 to 8.95) | -0.49% (-0.54 to -0.43) | 7006 (4919 to 6205) | 7380 (5230 to 9569) | 55.36 (39.22 to 72.52) | -0.23% (-0.28 to -0.19) |
| Greenland | 17 (13 to 22) | 19 (15 to 26) | 31.96 (24.17 to 42.61) | -0.76% (-0.82 to -0.71) | 89 (65 to 0) | 91 (64 to 116) | 138.78 (98.2 to 176.26) | -0.78% (-0.82 to -0.74) |
| Grenada | 4 (3 to 4) | 5 (4 to 6) | 5.16 (4.23 to 6.22) | 0.42% (-0.09 to 0.93) | 37 (23 to 1404) | 48 (33 to 62) | 42.44 (29.95 to 56.07) | -0.46% (-0.55 to -0.37) |
| Guam | 11 (9 to 15) | 16 (13 to 21) | 9.48 (7.27 to 12.21) | -0.03% (-0.12 to 0.05) | 79 (57 to 22) | 119 (85 to 153) | 66.28 (47.68 to 85.47) | 0% (-0.09 to 0.09) |
| Guatemala | 871 (500 to 1724) | 1011 (825 to 1215) | 6.13 (4.96 to 7.38) | -1.07% (-1.51 to -0.63) | 9339 (3905 to 3514) | 10479 (6703 to 18661) | 69.11 (42.97 to 126.6) | -2.25% (-2.31 to -2.19) |
| Guinea | 475 (354 to 645) | 1043 (750 to 1460) | 11.26 (8.11 to 15.71) | 0.5% (0.2 to 0.8) | 3086 (2202 to 6333) | 6731 (4870 to 8518) | 76.25 (56.06 to 95.6) | 0.45% (0.35 to 0.54) |
| Guinea-Bissau | 92 (65 to 131) | 178 (124 to 255) | 12.12 (8.62 to 17.06) | -0.25% (-0.9 to 0.4) | 541 (393 to 1394) | 1184 (852 to 1523) | 83.21 (60.68 to 105.86) | 0.32% (0.24 to 0.4) |
| Guyana | 37 (31 to 44) | 46 (39 to 55) | 6.32 (5.24 to 7.51) | 0.43% (0.04 to 0.81) | 260 (189 to 1324) | 328 (235 to 416) | 42.51 (30.49 to 53.77) | 0.36% (0.32 to 0.41) |
| Haiti | 258 (210 to 315) | 505 (409 to 612) | 4.43 (3.6 to 5.33) | 0.61% (-3.09 to 4.46) | 1824 (1321 to 2635) | 18498 (8950 to 34403) | 157.72 (77.38 to 294.33) | 7.51% (5.59 to 9.47) |
| Honduras | 229 (186 to 276) | 511 (407 to 632) | 5.47 (4.37 to 6.73) | -0.78% (-2.64 to 1.12) | 1644 (1189 to 358) | 4760 (3333 to 6327) | 54.44 (38.4 to 72.21) | 0.61% (0.19 to 1.03) |
| Hungary | 2243 (1764 to 2836) | 1657 (1300 to 2100) | 13.54 (10.84 to 16.6) | -1.53% (-1.66 to -1.4) | 11949 (8547 to 2252) | 10581 (7521 to 13774) | 85.33 (60.17 to 112.29) | -0.67% (-0.83 to -0.51) |
| Iceland | 23 (19 to 29) | 35 (28 to 45) | 8.59 (6.84 to 10.75) | -0.4% (-0.79 to 0) | 152 (107 to 99) | 247 (175 to 324) | 60.05 (42.14 to 79.54) | 0.03% (-0.04 to 0.09) |
| India | 71333 (56267 to 91142) | 134888 (103757 to 173814) | 10.4 (8.02 to 13.35) | 0.14% (-0.01 to 0.28) | 425838 (312874 to 81) | 887447 (644356 to 1112943) | 65.92 (48.07 to 82.32) | 0.24% (0 to 0.48) |
| Indonesia | 18250 (14109 to 23763) | 25244 (19349 to 32889) | 10.56 (8.04 to 13.72) | -0.69% (-1.4 to 0.02) | 138111 (102139 to 65) | 210217 (153290 to 266273) | 78.53 (57.48 to 99.33) | -0.55% (-0.64 to -0.47) |
| Iran (Islamic Republic of) | 24800 (13327 to 45554) | 4164 (3373 to 5116) | 4.99 (4.08 to 6.07) | -2.34% (-4.22 to -0.42) | 58541 (29148 to 5157) | 58925 (36923 to 97182) | 63.33 (39.87 to 101.97) | -2.22% (-2.33 to -2.11) |
| Iraq | 1862 (1081 to 3507) | 3076 (2202 to 4940) | 7.21 (5.24 to 11.3) | 5.73% (2.43 to 9.14) | 59709 (18003 to 416) | 106419 (35020 to 295453) | 264.58 (89.56 to 729.17) | -0.79% (-1.21 to -0.37) |
| Ireland | 307 (247 to 379) | 454 (357 to 575) | 8.28 (6.59 to 10.35) | -0.19% (-0.32 to -0.05) | 1985 (1414 to 2959) | 3395 (2405 to 4422) | 58.92 (41.51 to 77.39) | 0.24% (0.14 to 0.34) |
| Israel | 396 (315 to 513) | 700 (560 to 870) | 7.16 (5.76 to 8.82) | -0.01% (-1.06 to 1.06) | 2599 (1868 to 1238) | 6892 (4598 to 10405) | 72.66 (47.88 to 111.2) | 1.32% (1.18 to 1.46) |
| Italy | 6984 (5309 to 9235) | 7114 (5225 to 9725) | 7.77 (6.07 to 9.8) | -1.69% (-1.98 to -1.39) | 40412 (28984 to 3273) | 44023 (31605 to 56655) | 52.32 (37.46 to 67.61) | -1.09% (-1.4 to -0.78) |
| Jamaica | 107 (85 to 138) | 124 (101 to 150) | 4.3 (3.52 to 5.18) | -0.09% (-0.23 to 0.04) | 786 (568 to 449) | 1076 (769 to 1376) | 36.01 (25.82 to 46.13) | -0.27% (-0.36 to -0.18) |
| Japan | 19707 (15505 to 24733) | 26116 (19727 to 35484) | 13.11 (10.3 to 16.7) | -0.38% (-0.47 to -0.29) | 141594 (100178 to 48) | 177770 (125301 to 229752) | 93.26 (65.6 to 121.17) | -0.12% (-0.23 to -0.01) |
| Jordan | 143 (118 to 173) | 456 (379 to 543) | 4.1 (3.39 to 4.91) | 0.28% (-0.25 to 0.83) | 973 (690 to 339) | 3679 (2567 to 4814) | 34.42 (24.17 to 44.83) | -0.34% (-0.43 to -0.24) |
| Kazakhstan | 1384 (1137 to 1671) | 1558 (1281 to 1873) | 8.42 (6.92 to 10.08) | 0.3% (0.16 to 0.44) | 10394 (7403 to 39) | 11569 (8175 to 14962) | 60.05 (42.33 to 77.64) | 0.01% (-0.14 to 0.16) |
| Kenya | 858 (681 to 1054) | 1995 (1580 to 2483) | 4.88 (3.85 to 6.11) | 0.22% (-0.12 to 0.56) | 5256 (3826 to 2203) | 15246 (11075 to 20379) | 36.45 (26.51 to 47.19) | 0.75% (0.44 to 1.05) |
| Kiribati | 2 (2 to 3) | 3 (3 to 4) | 3.09 (2.53 to 3.81) | -0.95% (-1.75 to -0.15) | 13 (9 to 9235) | 24 (18 to 32) | 22.4 (16.17 to 28.94) | 0.18% (0.06 to 0.31) |
| Kuwait | 702 (234 to 1879) | 222 (183 to 267) | 4.97 (4.11 to 5.98) | -3.31% (-5.56 to -1.01) | 810 (547 to 6290) | 2655 (1727 to 4220) | 52.03 (34.5 to 79.55) | -1.46% (-1.86 to -1.06) |
| Kyrgyzstan | 327 (271 to 389) | 391 (322 to 474) | 6.02 (4.96 to 7.29) | -0.78% (-1.2 to -0.36) | 2318 (1672 to 3) | 3234 (2302 to 4195) | 51.94 (37.36 to 67.25) | -0.43% (-0.64 to -0.21) |
| Lao People's Democratic Republic | 305 (168 to 640) | 279 (221 to 352) | 4.24 (3.39 to 5.29) | -0.63% (-1.03 to -0.21) | 1114 (814 to 4822) | 2208 (1612 to 2785) | 33.9 (24.83 to 42.62) | -0.25% (-0.33 to -0.17) |
| Latvia | 527 (423 to 652) | 259 (209 to 320) | 11.7 (9.51 to 14.25) | -1.79% (-1.95 to -1.63) | 3483 (2489 to 3941) | 1823 (1280 to 2382) | 74.56 (51.91 to 98.11) | -1.54% (-1.71 to -1.38) |
| Lebanon | 547 (234 to 1362) | 244 (201 to 294) | 4.7 (3.88 to 5.67) | -1.18% (-2.51 to 0.18) | 10077 (2378 to 68) | 7227 (2416 to 20570) | 135.04 (45.07 to 386.42) | -3.04% (-3.08 to -3) |
| Lesotho | 123 (97 to 155) | 184 (142 to 241) | 9.66 (7.48 to 12.71) | 0.83% (0.66 to 0.99) | 788 (562 to 851) | 984 (707 to 1254) | 49.82 (36.14 to 63.11) | -0.36% (-0.81 to 0.08) |
| Liberia | 1272 (356 to 3721) | 339 (250 to 461) | 9.46 (7.03 to 12.76) | -4.62% (-6.67 to -2.53) | 1189 (810 to 8) | 3991 (2373 to 7994) | 105.08 (63.46 to 202.71) | 0.35% (-0.23 to 0.92) |
| Libya | 186 (150 to 231) | 815 (471 to 1647) | 11.94 (6.81 to 24.42) | 4.35% (1.65 to 7.11) | 2072 (1260 to 1500) | 7366 (3820 to 15730) | 100.12 (52.04 to 214.74) | 1.73% (1.22 to 2.25) |
| Lithuania | 659 (531 to 815) | 448 (358 to 555) | 13.21 (10.75 to 16.16) | -0.8% (-0.93 to -0.66) | 4346 (3095 to 1671) | 3002 (2125 to 3902) | 82.01 (57.1 to 107.2) | -0.82% (-0.94 to -0.7) |
| Luxembourg | 46 (37 to 58) | 77 (59 to 100) | 9.96 (7.89 to 12.57) | -0.45% (-0.51 to -0.39) | 295 (209 to 658) | 495 (353 to 644) | 63.52 (44.98 to 82.68) | -0.22% (-0.29 to -0.15) |
| Madagascar | 1106 (812 to 1516) | 2400 (1784 to 3217) | 10.99 (8.23 to 14.84) | -0.14% (-0.23 to -0.05) | 7170 (5120 to 3507) | 17061 (12159 to 21638) | 82.2 (58.71 to 103.81) | -0.11% (-0.25 to 0.03) |
| Malawi | 750 (563 to 1004) | 1498 (1148 to 1968) | 10.41 (7.95 to 13.73) | -0.07% (-0.21 to 0.07) | 4380 (3111 to 1004) | 9618 (6811 to 12315) | 69.76 (49.97 to 87.48) | 0.7% (0.37 to 1.02) |
| Malaysia | 742 (575 to 955) | 1647 (1281 to 2113) | 5.4 (4.25 to 6.88) | 0.12% (-0.09 to 0.34) | 5366 (3824 to 340) | 11627 (8356 to 15152) | 36.38 (26.1 to 47.31) | -0.28% (-0.55 to -0.01) |
| Maldives | 7 (6 to 8) | 19 (16 to 23) | 4.18 (3.43 to 5.08) | 0.13% (-1.3 to 1.58) | 44 (32 to 4022) | 164 (116 to 219) | 31.96 (22.69 to 41.99) | 0.46% (0.28 to 0.65) |
| Mali | 832 (585 to 1265) | 1931 (1389 to 2803) | 12.05 (8.84 to 16.52) | -0.75% (-1.94 to 0.46) | 4043 (2894 to 240) | 13001 (8825 to 20343) | 88.44 (62.02 to 137.71) | 1.04% (0.67 to 1.42) |
| Malta | 32 (26 to 40) | 51 (39 to 66) | 9.14 (7.24 to 11.56) | 0.36% (0.3 to 0.43) | 232 (164 to 29) | 370 (261 to 483) | 65.16 (45.86 to 86.43) | 0.78% (0.67 to 0.89) |
| Marshall Islands | 1 (1 to 2) | 2 (2 to 3) | 4.29 (3.51 to 5.28) | 0.2% (0.15 to 0.26) | 9 (6 to 513) | 15 (11 to 20) | 28.38 (20.57 to 36.2) | 0.02% (-0.05 to 0.09) |
| Mauritania | 213 (150 to 304) | 383 (269 to 545) | 11.89 (8.46 to 16.79) | -0.17% (-0.38 to 0.04) | 1351 (979 to 14) | 2685 (1935 to 3434) | 88.96 (64.48 to 113.53) | 0% (-0.22 to 0.21) |
| Mauritius | 86 (66 to 113) | 140 (107 to 183) | 10 (7.71 to 13.01) | 0.67% (0.57 to 0.78) | 622 (444 to 1472) | 1072 (778 to 1385) | 69.48 (50.5 to 89.34) | 0.47% (0.37 to 0.58) |
| Mexico | 12232 (9539 to 15778) | 17497 (13364 to 22764) | 14.31 (10.9 to 18.6) | 0.78% (0.37 to 1.18) | 83476 (60063 to 603) | 120381 (86536 to 154329) | 94.42 (67.82 to 121.07) | 1.3% (0.67 to 1.92) |
| Micronesia (Federated States of) | 4 (3 to 4) | 4 (3 to 5) | 4.53 (3.7 to 5.55) | 0.32% (-0.85 to 1.51) | 21 (15 to 138) | 29 (21 to 37) | 30.22 (21.83 to 38.78) | 0.44% (0.27 to 0.61) |
| Monaco | 8 (6 to 11) | 11 (8 to 16) | 19.89 (14.8 to 26.68) | 0.44% (0.39 to 0.49) | 47 (33 to 16) | 67 (47 to 88) | 131.68 (91.09 to 176.67) | 0.48% (0.39 to 0.56) |
| Mongolia | 147 (122 to 175) | 275 (226 to 330) | 8.06 (6.64 to 9.64) | 0.54% (0.21 to 0.86) | 951 (688 to 3) | 2105 (1497 to 2698) | 60.2 (42.74 to 76.81) | 0.49% (0.42 to 0.57) |
| Montenegro | 80 (65 to 97) | 86 (69 to 105) | 12.77 (10.37 to 15.45) | 0% (-0.06 to 0.07) | 587 (418 to 44) | 657 (462 to 856) | 88.24 (61.84 to 115.41) | -0.18% (-0.27 to -0.1) |
| Morocco | 1146 (938 to 1394) | 1836 (1507 to 2226) | 5.23 (4.28 to 6.35) | 0.05% (-0.24 to 0.34) | 10116 (7230 to 749) | 15820 (11427 to 20411) | 42.73 (30.86 to 54.97) | -0.43% (-0.47 to -0.39) |
| Mozambique | 1885 (1194 to 3430) | 3459 (2568 to 4656) | 15.78 (11.67 to 21.52) | 0.19% (-0.14 to 0.53) | 21792 (9745 to 4) | 20719 (14681 to 29422) | 101.89 (72.43 to 150.21) | -2.59% (-2.76 to -2.42) |
| Myanmar | 2114 (1669 to 2836) | 2798 (2239 to 3572) | 5.4 (4.32 to 6.85) | 0.42% (-1.86 to 2.76) | 18809 (10422 to 10235) | 33553 (21725 to 50386) | 60.75 (39.52 to 90.45) | 1.22% (0.64 to 1.81) |
| Namibia | 103 (79 to 135) | 202 (154 to 267) | 9.62 (7.3 to 12.82) | 0.15% (-0.11 to 0.41) | 1241 (695 to 307) | 1421 (1015 to 1872) | 68.15 (48.73 to 90.63) | -1.54% (-1.83 to -1.26) |
| Nauru | 1 (1 to 1) | 1 (1 to 1) | 10.34 (7.91 to 13.46) | 0.41% (0.33 to 0.49) | 4 (3 to 645) | 5 (4 to 7) | 58.44 (42 to 75.55) | 0.29% (0.15 to 0.42) |
| Nepal | 1039 (854 to 1257) | 2007 (1633 to 2454) | 7.11 (5.77 to 8.76) | 0.37% (-0.91 to 1.65) | 6953 (5034 to 4) | 18091 (12597 to 24724) | 64.05 (45.27 to 86.98) | 1.17% (1.04 to 1.3) |
| Netherlands | 1306 (1026 to 1627) | 2465 (1825 to 3305) | 9.43 (7.36 to 12.06) | 0.6% (0.21 to 0.98) | 8074 (5744 to 155) | 11974 (8526 to 15625) | 51.99 (36.97 to 68.28) | 0.16% (-0.06 to 0.39) |
| New Zealand | 539 (439 to 658) | 850 (675 to 1072) | 15.87 (12.83 to 19.48) | 0.1% (0.04 to 0.17) | 3693 (2583 to 104) | 5845 (4127 to 7620) | 108.93 (77.24 to 142.9) | 0.05% (-0.1 to 0.2) |
| Nicaragua | 210 (162 to 286) | 292 (240 to 353) | 4.81 (3.92 to 5.8) | -0.92% (-2.2 to 0.37) | 10677 (3015 to 10682) | 7105 (3061 to 17891) | 121.81 (51.4 to 308.78) | -2.85% (-2.92 to -2.78) |
| Niger | 626 (467 to 851) | 1736 (1306 to 2329) | 11.17 (8.42 to 14.95) | 0.27% (0.1 to 0.44) | 3532 (2571 to 1627) | 10196 (7356 to 12935) | 72.66 (52.93 to 91.5) | 0.16% (0.04 to 0.28) |
| Nigeria | 6673 (5070 to 8794) | 16736 (12504 to 22446) | 10.67 (7.95 to 14.37) | 0.65% (0.45 to 0.85) | 41034 (29577 to 1362) | 107235 (77703 to 141553) | 69.23 (49.79 to 88.71) | 0.57% (0.3 to 0.84) |
| Niue | 0 (0 to 0) | 0 (0 to 0) | 10.19 (7.77 to 13.2) | 0.59% (-0.25 to 1.44) | 1 (1 to 13) | 1 (1 to 1) | 62.07 (44.58 to 80.64) | 0.72% (0.61 to 0.83) |
| North Macedonia | 197 (160 to 240) | 247 (197 to 301) | 11.03 (8.89 to 13.43) | 0.46% (0.26 to 0.65) | 1697 (1189 to 978) | 2107 (1487 to 2725) | 80.53 (56.54 to 104.74) | 0.02% (-0.09 to 0.14) |
| Northern Mariana Islands | 5 (4 to 6) | 6 (4 to 7) | 13.32 (10.13 to 17.5) | 0.12% (-0.03 to 0.28) | 29 (21 to 6) | 36 (26 to 47) | 70.3 (50.8 to 91.08) | -0.18% (-0.28 to -0.09) |
| Norway | 1525 (1067 to 2203) | 2129 (1430 to 3202) | 25.97 (18.45 to 37.37) | -0.16% (-0.2 to -0.12) | 6642 (4671 to 276) | 9426 (6643 to 12304) | 134.45 (93.93 to 176.77) | 0.03% (-0.06 to 0.11) |
| Oman | 111 (87 to 142) | 279 (226 to 355) | 6.82 (5.44 to 8.53) | -0.1% (-0.29 to 0.1) | 739 (530 to 10987) | 2116 (1481 to 2770) | 44.37 (31.18 to 57.6) | -0.34% (-0.4 to -0.27) |
| Pakistan | 4392 (3396 to 5692) | 11294 (8483 to 15235) | 5.67 (4.25 to 7.62) | 1.39% (0.32 to 2.47) | 28874 (21063 to 14) | 93040 (65998 to 127413) | 50.51 (36.13 to 67.48) | 1.6% (1.39 to 1.82) |
| Palau | 2 (1 to 2) | 3 (2 to 4) | 16.73 (12.8 to 21.82) | 0.44% (0.4 to 0.48) | 10 (7 to 131) | 17 (12 to 22) | 78.35 (55.71 to 101.24) | 0.18% (0.15 to 0.22) |
| Palestine | 357 (142 to 926) | 254 (207 to 324) | 5.41 (4.41 to 6.8) | -1.87% (-5.86 to 2.3) | 5353 (1742 to 1227) | 13321 (4255 to 37063) | 308.99 (100.23 to 843.44) | 1.21% (0.79 to 1.63) |
| Panama | 125 (101 to 154) | 218 (176 to 265) | 5.22 (4.24 to 6.34) | -0.35% (-0.46 to -0.25) | 1175 (776 to 8794) | 1936 (1346 to 2667) | 46.09 (32.02 to 63.37) | -0.55% (-0.62 to -0.47) |
| Papua New Guinea | 154 (122 to 200) | 414 (337 to 509) | 5.2 (4.19 to 6.39) | -0.45% (-1.89 to 1.01) | 882 (641 to 127) | 2956 (2106 to 3811) | 35.96 (25.8 to 45.93) | 0.71% (0.39 to 1.02) |
| Paraguay | 188 (155 to 226) | 359 (293 to 439) | 5.25 (4.3 to 6.41) | 0.22% (0.13 to 0.31) | 1494 (1070 to 1274) | 2818 (2022 to 3605) | 41.84 (30.33 to 53.21) | -0.35% (-0.45 to -0.25) |
| Peru | 2220 (1205 to 4832) | 1473 (1223 to 1748) | 4.35 (3.61 to 5.15) | -1.8% (-2.59 to -0.99) | 11805 (6784 to 7097) | 15903 (10527 to 25226) | 46.34 (30.77 to 73.26) | -1.13% (-1.21 to -1.06) |
| Philippines | 4386 (3208 to 6290) | 4962 (3991 to 6137) | 4.75 (3.8 to 5.86) | -0.42% (-1.24 to 0.41) | 26715 (16967 to 2984) | 43970 (30346 to 63181) | 42.64 (29.62 to 61.04) | -0.79% (-0.88 to -0.71) |
| Poland | 5733 (4558 to 7097) | 5981 (4676 to 7524) | 13.38 (10.64 to 16.56) | -0.35% (-0.4 to -0.3) | 40801 (29267 to 24733) | 43601 (30773 to 56420) | 88.67 (62.53 to 115.2) | -0.44% (-0.57 to -0.32) |
| Portugal | 844 (690 to 1026) | 912 (716 to 1160) | 6.11 (4.96 to 7.43) | -1.15% (-1.24 to -1.06) | 6969 (4907 to 27) | 6283 (4459 to 8202) | 42.6 (29.89 to 56.04) | -1.51% (-1.58 to -1.43) |
| Puerto Rico | 452 (349 to 580) | 613 (459 to 804) | 13.28 (10.23 to 17.07) | 0.59% (0.13 to 1.06) | 3152 (2227 to 1) | 4044 (2883 to 5316) | 88.38 (62.27 to 116.48) | 0.05% (-0.01 to 0.12) |
| Qatar | 32 (26 to 39) | 204 (168 to 248) | 6.72 (5.53 to 8.09) | 0.02% (-0.11 to 0.14) | 255 (183 to 1476) | 1525 (1072 to 2024) | 46.7 (32.88 to 61.19) | -0.67% (-0.7 to -0.64) |
| Republic of Korea | 4182 (3485 to 4986) | 6124 (4942 to 7543) | 9.47 (7.7 to 11.51) | -0.34% (-0.45 to -0.24) | 28128 (19849 to 3) | 44931 (31441 to 58697) | 62 (43.42 to 80.82) | -0.29% (-0.37 to -0.22) |
| Republic of Moldova | 562 (454 to 702) | 381 (309 to 462) | 9.52 (7.8 to 11.57) | -1.35% (-1.78 to -0.92) | 4120 (2959 to 3453) | 3227 (2279 to 4171) | 70.89 (50.25 to 91.83) | -0.96% (-1.11 to -0.8) |
| Romania | 3310 (2715 to 3957) | 2425 (1962 to 2953) | 11.63 (9.47 to 14.12) | -0.7% (-0.83 to -0.56) | 26357 (18966 to 2433) | 19666 (14015 to 25656) | 82.07 (58.15 to 107.18) | -0.87% (-1.02 to -0.72) |
| Russian Federation | 24982 (19584 to 31842) | 21366 (16720 to 27153) | 13.3 (10.53 to 16.82) | -0.59% (-0.93 to -0.24) | 184998 (131132 to 2836) | 156702 (111813 to 202955) | 86.79 (61.88 to 112.19) | -0.6% (-0.74 to -0.45) |
| Rwanda | 1598 (867 to 3273) | 1281 (933 to 1771) | 12.46 (9.13 to 17.06) | -4.35% (-7.32 to -1.28) | 4886 (3490 to 39) | 25354 (11173 to 61782) | 241.67 (109.14 to 578.84) | 1.07% (-1.04 to 3.22) |
| Saint Kitts and Nevis | 4 (3 to 6) | 7 (5 to 9) | 12.05 (9.42 to 15.18) | 0.03% (-0.14 to 0.2) | 25 (18 to 580) | 50 (35 to 64) | 73.94 (52.38 to 95.69) | 0.26% (0.21 to 0.31) |
| Saint Lucia | 6 (5 to 8) | 9 (7 to 11) | 4.79 (3.89 to 5.83) | 0.1% (-0.2 to 0.41) | 42 (30 to 449) | 78 (57 to 100) | 38.89 (28.13 to 49.74) | 0.18% (0.15 to 0.21) |
| Saint Vincent and the Grenadines | 4 (4 to 5) | 6 (5 to 7) | 4.82 (3.95 to 5.71) | 0.31% (-0.11 to 0.72) | 32 (23 to 154) | 47 (33 to 60) | 37.58 (26.84 to 48.17) | 0.28% (0.24 to 0.33) |
| Samoa | 8 (6 to 11) | 9 (7 to 11) | 4.53 (3.69 to 5.58) | 0.46% (-1.52 to 2.47) | 37 (26 to 19) | 79 (54 to 108) | 41.87 (29.06 to 56.76) | 1.74% (1.31 to 2.17) |
| San Marino | 5 (4 to 6) | 9 (6 to 12) | 19.86 (14.84 to 26.65) | 0.34% (0.27 to 0.41) | 33 (23 to 231) | 58 (40 to 76) | 136.5 (95.23 to 180.59) | 0.36% (0.3 to 0.42) |
| Sao Tome and Principe | 10 (8 to 13) | 21 (16 to 28) | 13.11 (10.03 to 17.54) | 0.8% (0.75 to 0.85) | 67 (48 to 15) | 145 (103 to 185) | 88.3 (62.55 to 111.65) | 0.49% (0.4 to 0.58) |
| Saudi Arabia | 1214 (999 to 1476) | 4304 (3458 to 5275) | 11.67 (9.34 to 14.41) | 1.16% (1.03 to 1.29) | 8009 (5687 to 828) | 28027 (19946 to 36763) | 70.57 (50.14 to 92.18) | 0.17% (0.08 to 0.26) |
| Senegal | 578 (428 to 786) | 1219 (885 to 1673) | 10.62 (7.73 to 14.65) | 0.03% (-0.11 to 0.16) | 3421 (2430 to 1265) | 8308 (6023 to 10410) | 73.69 (53.84 to 92.1) | 0.41% (0.35 to 0.46) |
| Serbia | 1074 (868 to 1324) | 1082 (873 to 1327) | 11.33 (9.16 to 13.75) | -1.18% (-2.62 to 0.27) | 9405 (6815 to 961) | 10185 (7050 to 14487) | 98.36 (67.04 to 141.44) | 0.33% (0.08 to 0.57) |
| Seychelles | 6 (5 to 8) | 11 (8 to 14) | 10.17 (7.77 to 13.29) | 0.14% (-0.07 to 0.35) | 43 (31 to 8) | 81 (58 to 104) | 69.62 (49.93 to 89.26) | 0.08% (0 to 0.17) |
| Sierra Leone | 280 (207 to 382) | 667 (479 to 929) | 10.74 (7.81 to 14.92) | -2.56% (-4.28 to -0.81) | 1766 (1285 to 87) | 6286 (4171 to 10476) | 100.72 (67.18 to 166.86) | 1.12% (0.51 to 1.74) |
| Singapore | 230 (191 to 276) | 418 (340 to 504) | 6.91 (5.63 to 8.34) | -0.42% (-0.5 to -0.33) | 1697 (1193 to 1106) | 3881 (2727 to 5081) | 53.38 (37.38 to 69.92) | -0.05% (-0.12 to 0.03) |
| Slovakia | 870 (701 to 1066) | 928 (735 to 1145) | 14.74 (11.84 to 18.04) | -0.31% (-0.43 to -0.18) | 5560 (3928 to 5) | 6544 (4662 to 8499) | 94.69 (67.43 to 123.15) | -0.28% (-0.39 to -0.16) |
| Slovenia | 411 (329 to 510) | 466 (364 to 597) | 16.56 (13.28 to 20.49) | -0.23% (-0.38 to -0.08) | 2596 (1853 to 91142) | 3019 (2147 to 3976) | 108.74 (76.76 to 143.08) | 0.19% (0.02 to 0.37) |
| Solomon Islands | 13 (11 to 16) | 31 (25 to 37) | 5.77 (4.72 to 7.01) | 0.71% (0.06 to 1.37) | 75 (54 to 1531) | 175 (126 to 225) | 32.33 (23.17 to 41.34) | 0.24% (0.15 to 0.33) |
| Somalia | 1386 (691 to 3196) | 2192 (1537 to 3307) | 13.38 (9.63 to 19.81) | 0.03% (-1.26 to 1.34) | 5141 (2845 to 1058) | 16214 (10017 to 30965) | 105.56 (67.24 to 192.4) | 0.05% (-0.16 to 0.26) |
| South Africa | 3579 (2694 to 4822) | 4748 (3614 to 6370) | 8.82 (6.74 to 11.72) | -0.41% (-0.64 to -0.18) | 24873 (17886 to 640) | 31489 (22407 to 39715) | 56.9 (40.53 to 71.47) | -1.06% (-1.36 to -0.76) |
| South Sudan | 550 (404 to 749) | 964 (698 to 1429) | 12.73 (9.33 to 17.57) | 0.7% (-0.66 to 2.09) | 5006 (3293 to 292) | 9635 (5876 to 18305) | 136.45 (86.68 to 247.7) | 0.8% (0.59 to 1) |
| Spain | 3241 (2655 to 3941) | 4547 (3526 to 5853) | 7.71 (6.12 to 9.56) | -0.21% (-0.33 to -0.08) | 23321 (16596 to 1724) | 34473 (24458 to 44862) | 56.71 (39.73 to 74.77) | 0.03% (-0.1 to 0.16) |
| Sri Lanka | 4222 (1659 to 10682) | 1496 (1195 to 1878) | 6.66 (5.34 to 8.41) | -3.83% (-6.11 to -1.5) | 10662 (6172 to 577) | 24070 (12260 to 51620) | 101.79 (51.42 to 221.79) | 1.44% (1.04 to 1.83) |
| Sudan | 3034 (1646 to 6333) | 3305 (2618 to 4167) | 9.07 (7.17 to 11.52) | -1.12% (-1.91 to -0.32) | 12460 (8534 to 0) | 31938 (20436 to 55444) | 96.49 (63.06 to 164.23) | 0.75% (0.61 to 0.9) |
| Suriname | 16 (13 to 21) | 28 (23 to 35) | 4.95 (4.04 to 6.05) | 0.47% (0.43 to 0.51) | 160 (108 to 83656) | 249 (178 to 326) | 40.88 (29.28 to 53.67) | -0.27% (-0.34 to -0.21) |
| Sweden | 2789 (1953 to 4022) | 3946 (2665 to 5921) | 24.06 (17.29 to 34.08) | 0.02% (-0.02 to 0.07) | 14031 (9879 to 4986) | 18973 (13417 to 24808) | 138.79 (96.32 to 184.22) | 0.11% (-0.02 to 0.23) |
| Switzerland | 1194 (929 to 1527) | 1460 (1091 to 1923) | 11.51 (8.97 to 14.54) | -1.02% (-1.14 to -0.9) | 6683 (4699 to 113) | 8610 (6048 to 11261) | 73.59 (51.43 to 97.32) | -0.7% (-0.93 to -0.47) |
| Syrian Arab Republic | 474 (388 to 577) | 2762 (1061 to 7102) | 25.67 (8.84 to 69.24) | 14.67% (9.88 to 19.68) | 6334 (2931 to 1153) | 56665 (16102 to 159728) | 403.19 (112.58 to 1150.34) | 6.57% (4.16 to 9.04) |
| Taiwan (Province of China) | 990 (800 to 1238) | 1177 (934 to 1463) | 3.87 (3.11 to 4.83) | -1.36% (-1.98 to -0.73) | 6119 (4255 to 97) | 8089 (5746 to 10379) | 24.99 (17.67 to 32.22) | -0.95% (-1.03 to -0.87) |
| Tajikistan | 351 (292 to 416) | 498 (414 to 595) | 5.34 (4.42 to 6.39) | -4.73% (-7.23 to -2.16) | 2367 (1683 to 1785) | 7748 (4353 to 15772) | 87.79 (49.52 to 174.94) | -0.02% (-0.96 to 0.93) |
| Thailand | 3095 (2432 to 3953) | 4088 (3294 to 5010) | 5.09 (4.14 to 6.32) | -0.39% (-0.84 to 0.05) | 23282 (16627 to 45554) | 31982 (23052 to 41443) | 36.05 (25.7 to 47.01) | -0.73% (-0.79 to -0.66) |
| Timor-Leste | 142 (50 to 380) | 46 (37 to 57) | 3.9 (3.15 to 4.85) | -7.99% (-10.59 to -5.32) | 1261 (429 to 2) | 1479 (569 to 3897) | 143.48 (54.81 to 376.86) | -0.55% (-1.13 to 0.04) |
| Togo | 295 (211 to 412) | 716 (511 to 1016) | 11.4 (8.31 to 15.7) | 0.17% (-0.03 to 0.37) | 1867 (1351 to 5) | 4915 (3518 to 6243) | 78.82 (56.91 to 99.46) | 0.11% (-0.11 to 0.33) |
| Tokelau | 0 (0 to 0) | 0 (0 to 0) | 8.98 (6.86 to 11.66) | 0.69% (0.61 to 0.77) | 1 (1 to 3721) | 1 (1 to 1) | 60.07 (43.1 to 77.17) | 0.4% (0.3 to 0.5) |
| Tonga | 3 (3 to 4) | 3 (3 to 4) | 3.54 (2.9 to 4.29) | 0.05% (-0.68 to 0.77) | 20 (14 to 4) | 25 (18 to 32) | 27.32 (19.52 to 35.51) | 0% (-0.21 to 0.21) |
| Trinidad and Tobago | 65 (48 to 99) | 64 (53 to 79) | 4.53 (3.68 to 5.55) | -0.17% (-0.38 to 0.04) | 390 (278 to 8) | 606 (436 to 779) | 37.81 (27.09 to 49.1) | 0.43% (0.34 to 0.52) |
| Tunisia | 365 (295 to 449) | 574 (467 to 697) | 4.89 (3.99 to 5.95) | 0.49% (0.36 to 0.62) | 2730 (1975 to 17) | 4717 (3406 to 6139) | 36.74 (26.46 to 47.62) | -0.26% (-0.33 to -0.19) |
| Turkey | 2725 (2128 to 3708) | 4391 (3619 to 5398) | 5.26 (4.34 to 6.47) | -0.65% (-2.12 to 0.83) | 18251 (13258 to 23763) | 40162 (27330 to 56767) | 43.68 (29.68 to 61.85) | 0.54% (0.27 to 0.82) |
| Turkmenistan | 208 (173 to 249) | 285 (237 to 341) | 5.59 (4.65 to 6.68) | -0.84% (-1.58 to -0.1) | 1498 (1072 to 798) | 2455 (1756 to 3185) | 48.12 (34.51 to 62.16) | -0.18% (-0.31 to -0.05) |
| Tuvalu | 1 (0 to 1) | 1 (1 to 1) | 8.94 (6.9 to 11.51) | 0.52% (0.45 to 0.59) | 4 (3 to 1027) | 6 (5 to 8) | 56.26 (40.61 to 72.18) | 0.31% (0.22 to 0.4) |
| Uganda | 1664 (1208 to 2433) | 3506 (2626 to 4657) | 11.22 (8.37 to 15.03) | -0.33% (-0.67 to 0.01) | 18110 (8333 to 389) | 26128 (18352 to 37337) | 93.1 (65.12 to 134.33) | -0.9% (-1.15 to -0.65) |
| Ukraine | 8330 (6687 to 10418) | 6116 (4927 to 7659) | 13.09 (10.6 to 16.3) | -0.55% (-0.87 to -0.23) | 61912 (43941 to 1482) | 44214 (31275 to 57074) | 81.82 (58.29 to 105.72) | -0.97% (-1.24 to -0.7) |
| United Arab Emirates | 102 (82 to 127) | 562 (454 to 695) | 5.64 (4.58 to 7) | -0.25% (-0.38 to -0.12) | 880 (633 to 4) | 5464 (3826 to 7080) | 42.93 (30.86 to 54.84) | -0.56% (-0.68 to -0.45) |
| United Kingdom | 6323 (4968 to 8043) | 9096 (6830 to 12284) | 10.18 (8.02 to 13.02) | 0.21% (0.16 to 0.27) | 41311 (29390 to 5692) | 55158 (39439 to 71101) | 65.14 (46.24 to 84.85) | 0.14% (0.06 to 0.22) |
| United Republic of Tanzania | 2217 (1681 to 2959) | 4873 (3700 to 6437) | 10.93 (8.26 to 14.53) | 0.03% (-0.05 to 0.11) | 13056 (9286 to 7) | 32929 (23333 to 41503) | 78.41 (55.6 to 98.96) | 0.66% (0.35 to 0.97) |
| United States of America | 64655 (50213 to 83656) | 108549 (80631 to 149208) | 24.1 (18.43 to 31.94) | -0.08% (-0.27 to 0.12) | 366838 (261078 to 500) | 515833 (364639 to 661820) | 120.29 (84.92 to 155.02) | -0.76% (-1.13 to -0.39) |
| United States Virgin Islands | 11 (8 to 14) | 13 (10 to 17) | 11.08 (8.71 to 13.96) | 0.03% (-0.14 to 0.19) | 84 (61 to 382) | 88 (63 to 113) | 69.89 (50.12 to 89.87) | -0.73% (-0.86 to -0.59) |
| Uruguay | 275 (221 to 340) | 322 (260 to 391) | 8.52 (6.93 to 10.37) | -0.1% (-0.18 to -0.02) | 2159 (1561 to 200) | 2499 (1787 to 3231) | 64.05 (45.78 to 83.38) | -0.24% (-0.33 to -0.15) |
| Uzbekistan | 1249 (1049 to 1482) | 2119 (1744 to 2552) | 6.33 (5.2 to 7.64) | 0.02% (-0.08 to 0.12) | 8854 (6375 to 352) | 16425 (11580 to 21093) | 49.02 (34.72 to 62.77) | -0.2% (-0.3 to -0.09) |
| Vanuatu | 4 (4 to 6) | 10 (8 to 12) | 3.79 (3.08 to 4.65) | -0.72% (-2.7 to 1.3) | 34 (24 to 1026) | 84 (59 to 112) | 33.36 (23.71 to 44.39) | 0.45% (-0.02 to 0.93) |
| Venezuela (Bolivarian Republic of) | 1184 (951 to 1472) | 1736 (1400 to 2127) | 6.23 (5.03 to 7.6) | -0.72% (-2.01 to 0.58) | 8941 (6413 to 926) | 15660 (11076 to 20541) | 52.79 (37.49 to 69.26) | -0.09% (-0.3 to 0.12) |
| Viet Nam | 2831 (2271 to 3514) | 5883 (4660 to 7367) | 6.19 (4.91 to 7.71) | 0.63% (0.42 to 0.85) | 19083 (14045 to 10418) | 41271 (29846 to 53245) | 39.15 (28.39 to 50.39) | 0.3% (0.24 to 0.36) |
| Yemen | 495 (404 to 603) | 8763 (2941 to 24072) | 25.95 (9.26 to 70.26) | 6.47% (4.12 to 8.86) | 6487 (3619 to 412) | 27471 (12186 to 66369) | 100.91 (45.87 to 237.91) | 0.58% (-0.13 to 1.29) |
| Zambia | 612 (472 to 798) | 1463 (1123 to 1917) | 10.83 (8.31 to 14.16) | 0.15% (0.08 to 0.22) | 3375 (2402 to 132131) | 9038 (6509 to 11379) | 66.11 (47.78 to 83.11) | 0.77% (0.35 to 1.2) |
| Zimbabwe | 659 (524 to 828) | 1164 (911 to 1469) | 9.82 (7.72 to 12.52) | -0.03% (-0.23 to 0.18) | 3920 (2824 to 3957) | 6175 (4495 to 7836) | 50.66 (36.64 to 63.58) | 0.19% (-0.44 to 0.82) |
