# Supplementary figures and images for: Spinal cord injury: global burden from 1990 to 2019 and projections up to 2030 using Bayesian age-period-cohort analysis
Source: Front Neurol. 2023 Dec 5;14:1304153. doi: 10.3389/fneur.2023.1304153 (PMC10729761; doi:10.3389/fneur.2023.1304153)

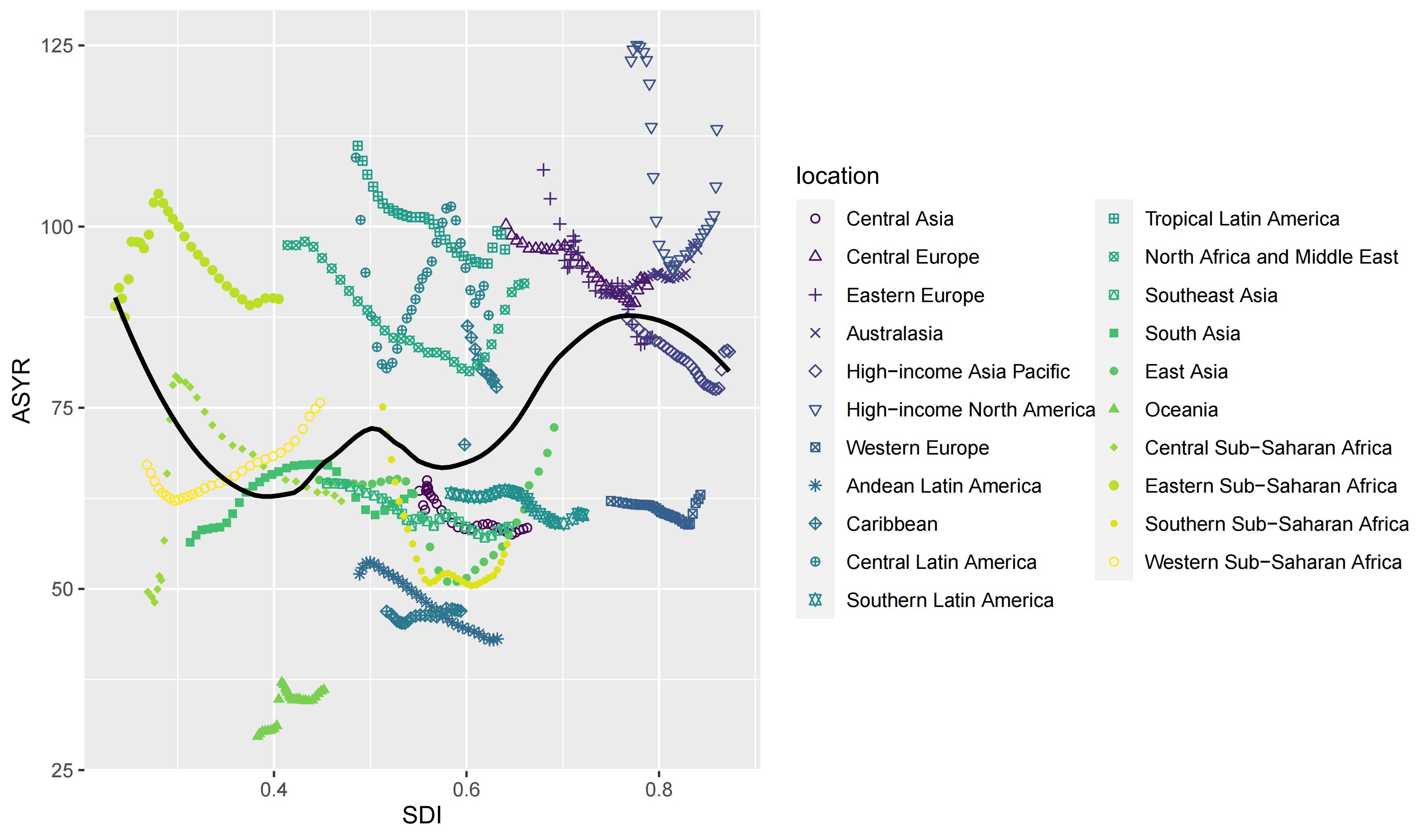

Supplement: Supplementary file 3 [file Image_1.jpeg]

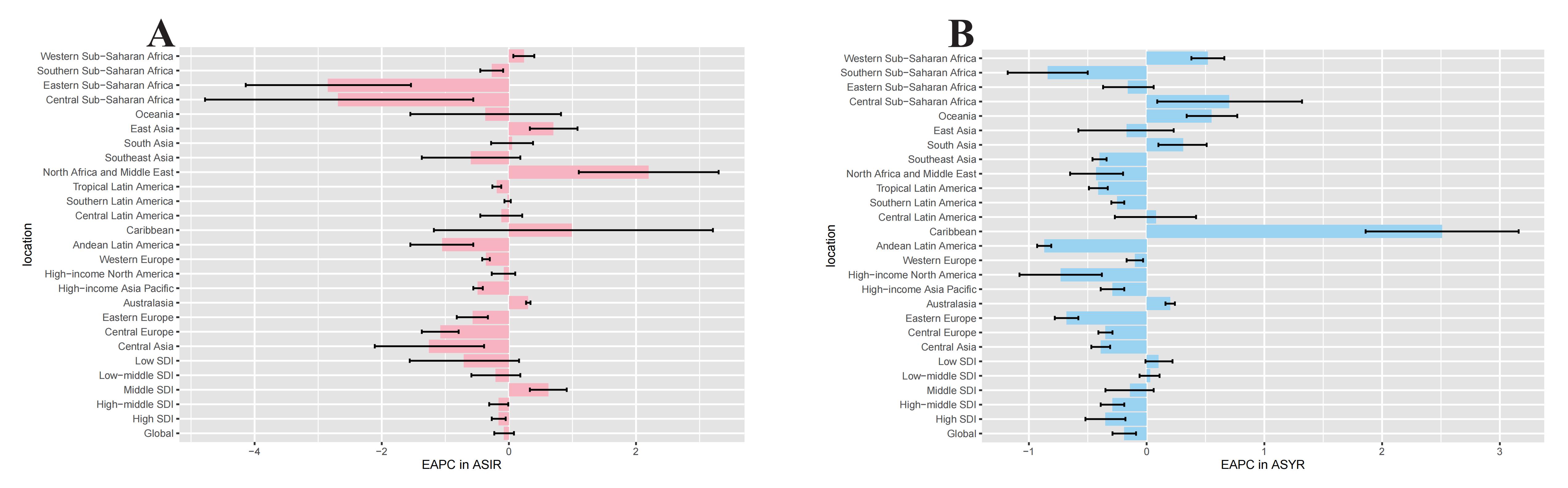

Supplement: Supplementary file 4 [file Image_2.jpeg]

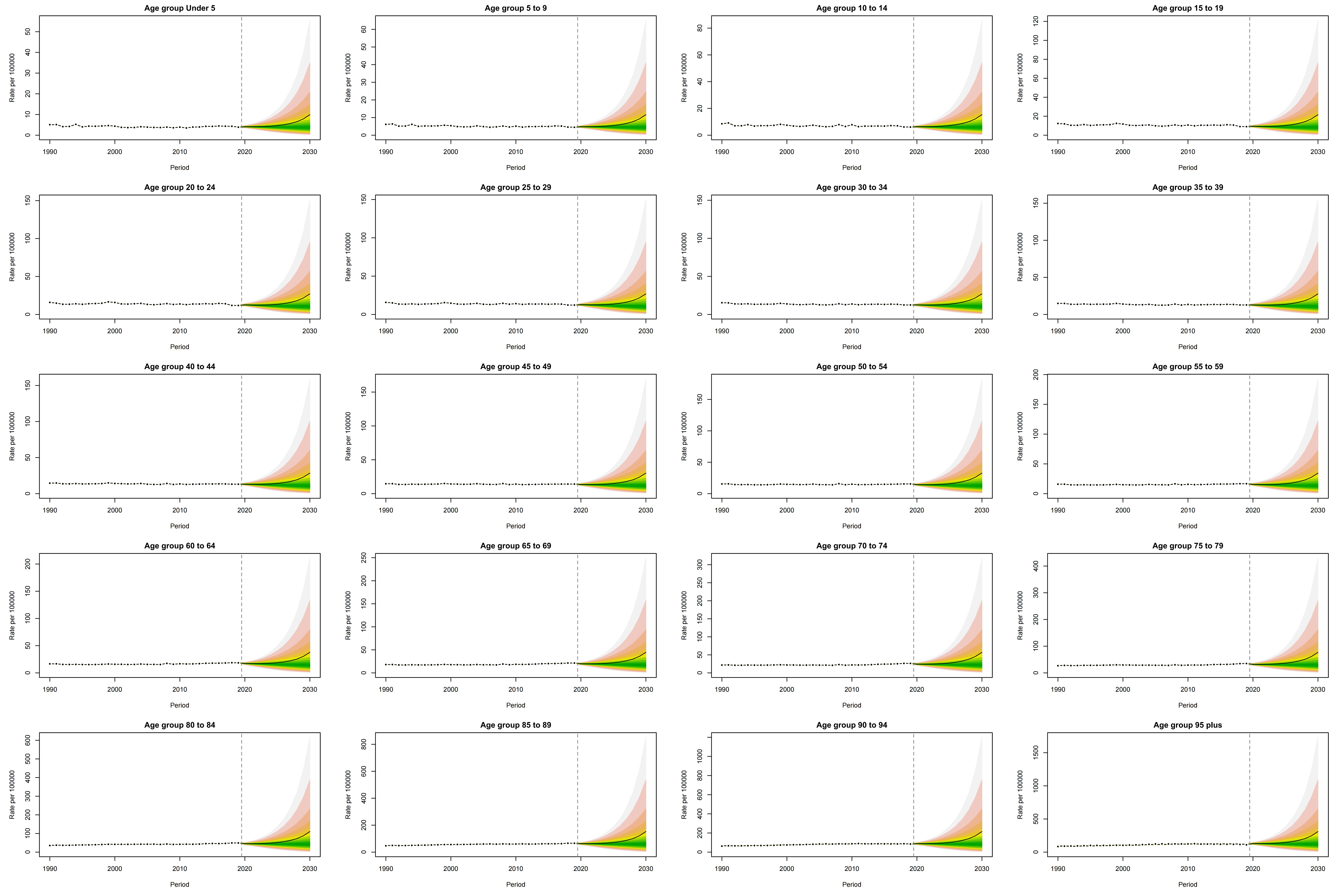

Supplement: Supplementary file 5 [file Image_3.jpeg]

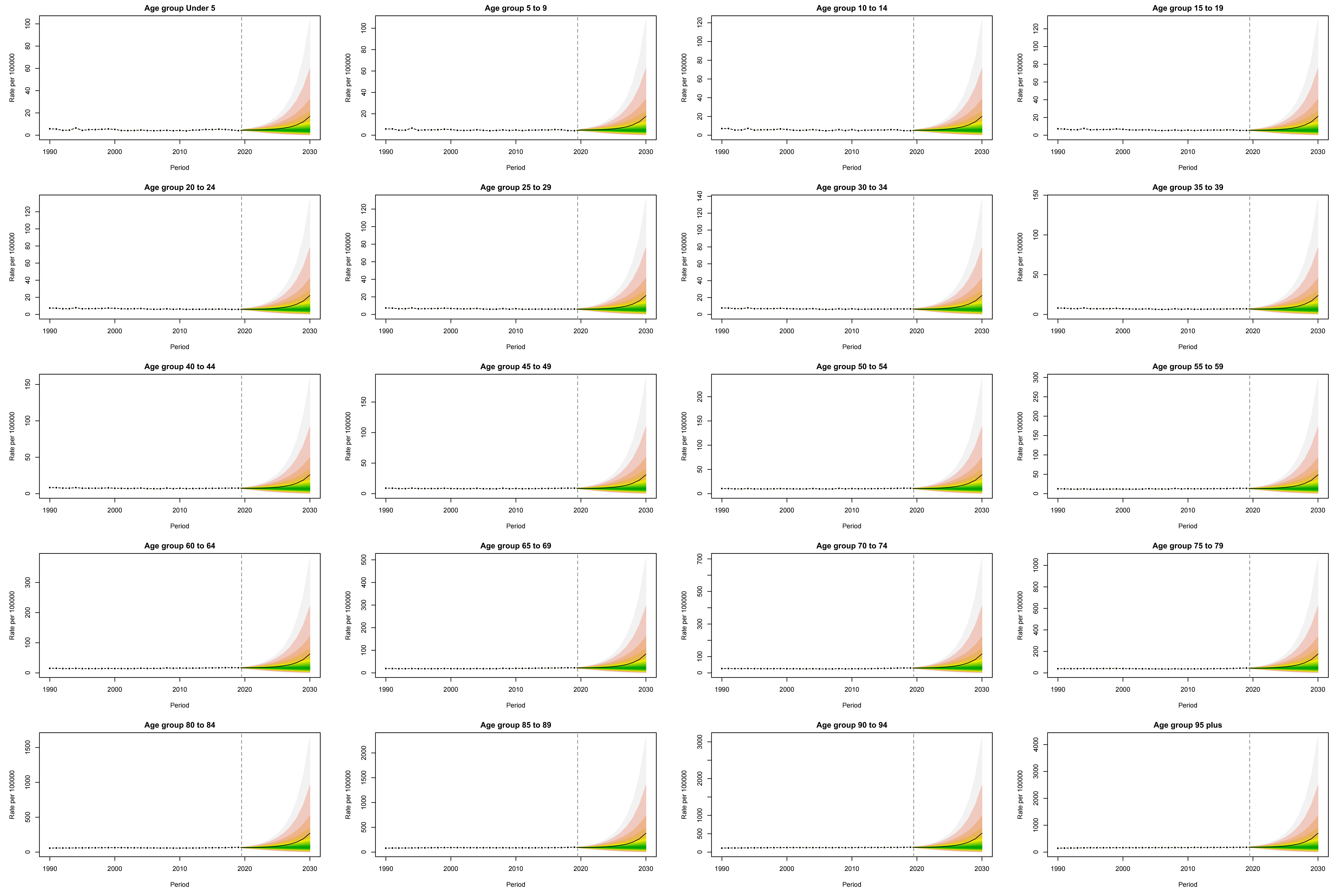

Supplement: Supplementary file 6 [file Image_4.jpeg]

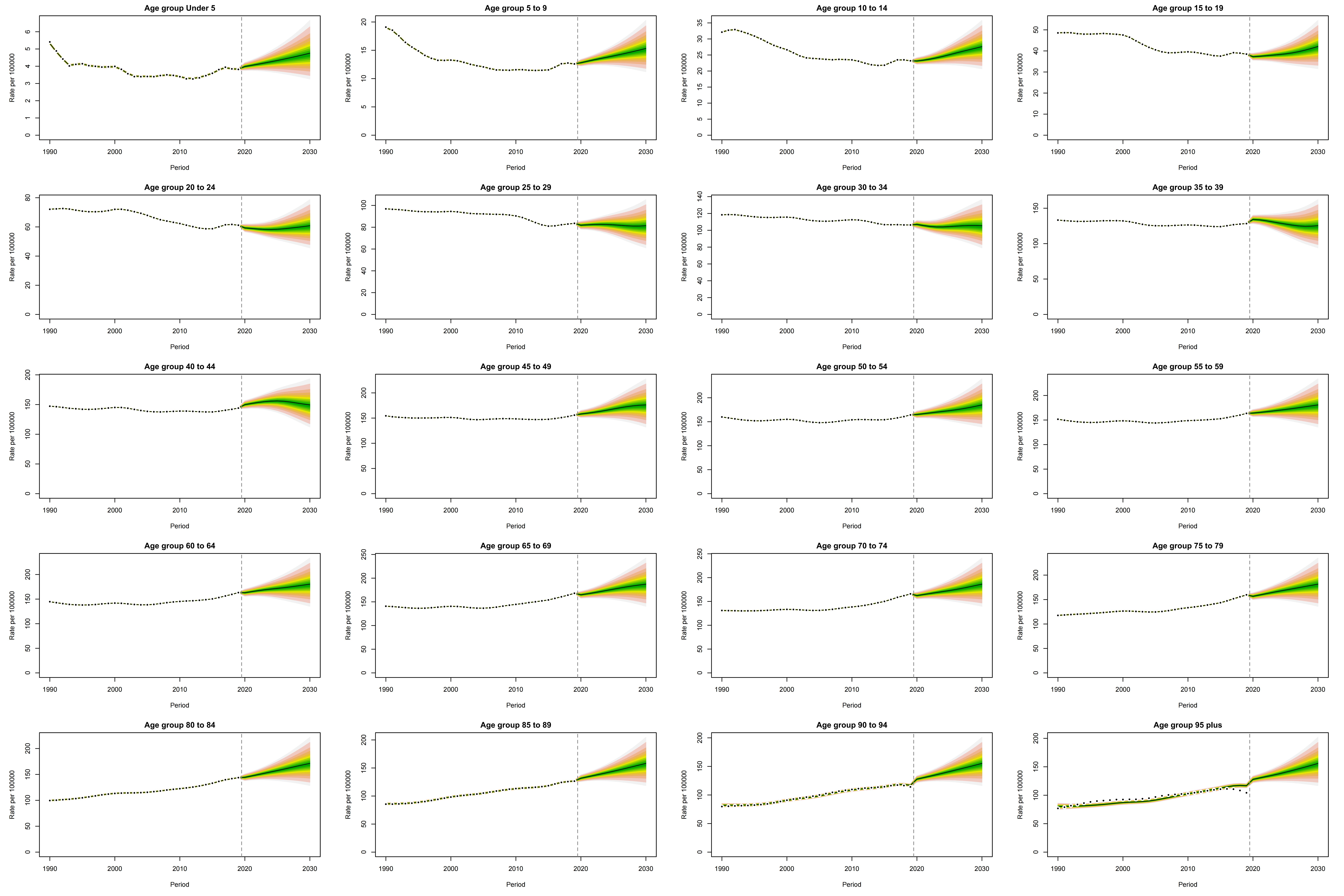

Supplement: Supplementary file 7 [file Image_5.jpeg]

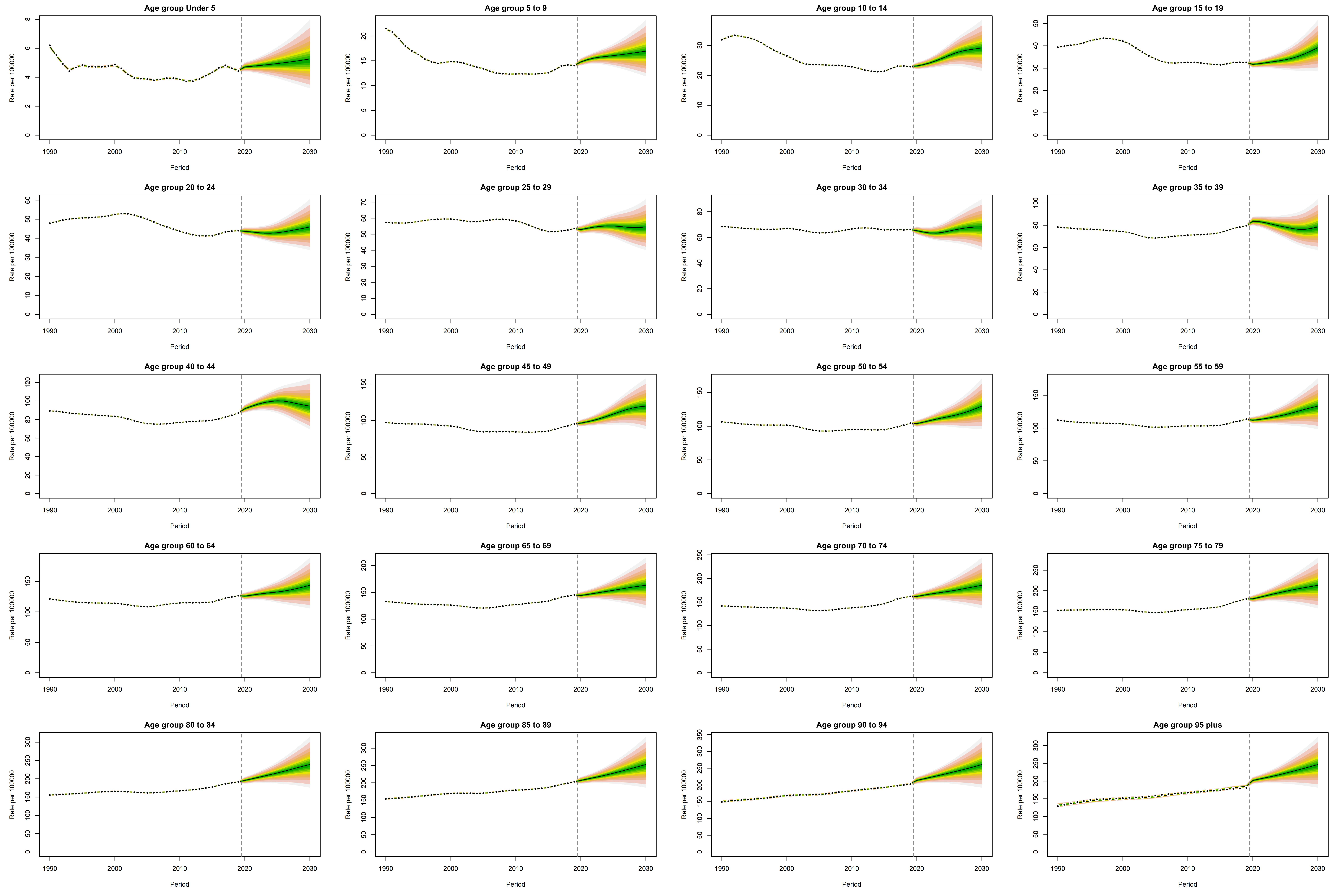

Supplement: Supplementary file 8 [file Image_6.jpeg]
